# Temporally Reconfigurable Reservoir Computing with Flexible Electrolyte‐Gated TFTs for High‐Performance Neuromorphic Processing

**DOI:** 10.1002/adma.202507979

**Published:** 2025-09-17

**Authors:** Kang Hyun Lee, Seohak Park, Mingu Kang, Jungyeop Oh, Wonbae Ahn, Hyeonji Lee, Seungsun Yoo, Hyunmin Kim, Min Kyu Lee, Sung‐Yool Choi

**Affiliations:** ^1^ Graduate School of Semiconductor Technology Korea Advanced Institute of Science and Technology (KAIST) 291 Daehak‐ro, Yuseong‐gu Daejeon 34141 Republic of Korea; ^2^ School of Electrical Engineering Korea Advanced Institute of Science and Technology (KAIST) 291 Daehak‐ro, Yuseong‐gu Daejeon 34141 Republic of Korea

**Keywords:** electrolyte‐gated transistors, flexible electronics, initiated chemical vapor deposition, medical image analysis, molybdenum disulfide, reservoir computing, spatiotemporal signal processing

## Abstract

Reservoir computing (RC), a brain‐inspired neuromorphic algorithm, offers simplicity and efficiency for processing spatiotemporal signals. However, conventional RC systems face limitations in handling diverse temporal scales and spatial complexities due to invariant temporal dynamics. This study introduces a temporally reconfigurable RC system utilizing ultrathin, flexible, all‐solid‐state electrolyte‐gated thin‐film transistors (UFLEX TFTs) with high performance: an on/off ratio of ≈10^7^, endurance beyond 2.5 × 10^4^ pulses, and low variability. UFLEX TFTs, based on molybdenum disulfide (MoS_2_) channels and organic–inorganic hybrid AlO_x_ dielectrics, enable modulation of temporal dynamics via simple electrical signals. The system maintains mechanical flexibility and robust performance after bending tests. By extracting features across varied temporal and spatial scales, it achieves classification accuracies of 90.3% for CIFAR‐10 object images and 81.8% for NIH chest X‐ray images. This work lays a foundation for flexible neuromorphic hardware systems capable of efficient, high‐performance spatiotemporal signal processing.

## Graphical abstract

A temporally reconfigurable reservoir computing system is developed using ultrathin, flexible, all‐solid‐state electrolyte‐gated thin‐film transistors (UFLEX TFTs). By simply tuning temporal dynamics electrically, the system achieves 93.7% accuracy for MNIST handwritten digits, 90.3% for CIFAR‐10 images, and 81.8% for chest X‐rays, surpassing CNNs in efficiency. Combining strong performance and flexibility, UFLEX TFTs enable neuromorphic hardware for next‐generation wearable edge devices.

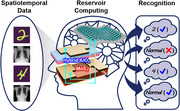

## 1 Introduction

The advancement of artificial intelligence (AI) has been significantly accelerated by three key factors: the continuous enhancement of computing performance driven by Moore's Law; the accumulation of vast amounts of data; and the development of high‐performance algorithms–establishing AI as a core driver of modern technology.

AI has demonstrated exceptional capabilities in tasks such as recognition and classification.^[^ ^1^ ^]^ Furthermore, innovations such as ChatGPT and DeepSeek for conversational AI, MidJourney for artistic image generation, and GitHub Copilot for programming code creation illustrate AI's human‐like creativity and intelligence.^[^ ^2^ ^]^ However, the high‐performance AI models behind these innovations involve highly complex architectures composed of vast networks of neurons and synapses, resulting in an exponential increase in the number of network parameters.^[^ ^3^ ^]^ This complexity demands substantial computational resources and energy consumption during both training and inference processes. Under the conventional von Neumann architecture, severe bottlenecks arise due to the frequent data transfer between memory and processing units, leading to significant energy inefficiency.^[^ ^3^, ^4^ ^]^ To address these challenges, lightweight neural networks have emerged as promising solutions.

Among various approaches, reservoir computing (RC) has emerged as a promising lightweight neural network model that replaces most complex recurrent structures with a largely static nonlinear reservoir.^[^ ^5^ ^]^ In RC, while inputs are projected into a high dimensional space by the reservoir, a readout layer processes the reservoir states. Notably, since only the readout layer undergoes training, the computational process is significantly simplified through methods such as backpropagation or linear regression.^[^ ^6^ ^]^ A key characteristic of the reservoir is its short‐term memory (STM), which enables it to retain recent inputs while discarding the influence of earlier ones. This property makes RC particularly suitable for identifying temporal pattern correlations.^[^ ^7^ ^]^ In recent years, advancements in theoretical models utilizing single nonlinear nodes^[^ ^7^, ^8^ ^]^ have facilitated the implementation of RC systems using electronic components such as memristors,^[^ ^9^ ^]^ ferroelectrics,^[^ ^10^ ^]^ and optical devices.^[^ ^11^ ^]^ These components offer advantages in terms of scalability, structural simplification, and improved energy efficiency.

Despite these advancements, the inherent temporal dynamics of the reservoir significantly influence performance and vary depending on the task.^[^ ^7^, ^12^ ^]^ Consequently, fine‐tuning of the reservoir is typically required to achieve optimal results. However, in most hardware‐based RC systems, the reservoir dynamics are inherently constrained by the fixed properties of the hardware, which are determined by the material composition and fabrication processes, thereby limiting them to a monotonic reservoir space.^[^ ^13^ ^]^ A potential solution is the development of devices with tunable reservoir dynamics, which have been experimentally demonstrated to efficiently extract information across multiple timescales within a single RC system.^[^ ^12^ ^]^ Thus, if the temporal dynamics of a single physical reservoir can be modulated across various timescales, it would enable effective application even in cases where prior knowledge of the optimal timescale for a given task is unavailable.

**Figure 1:**
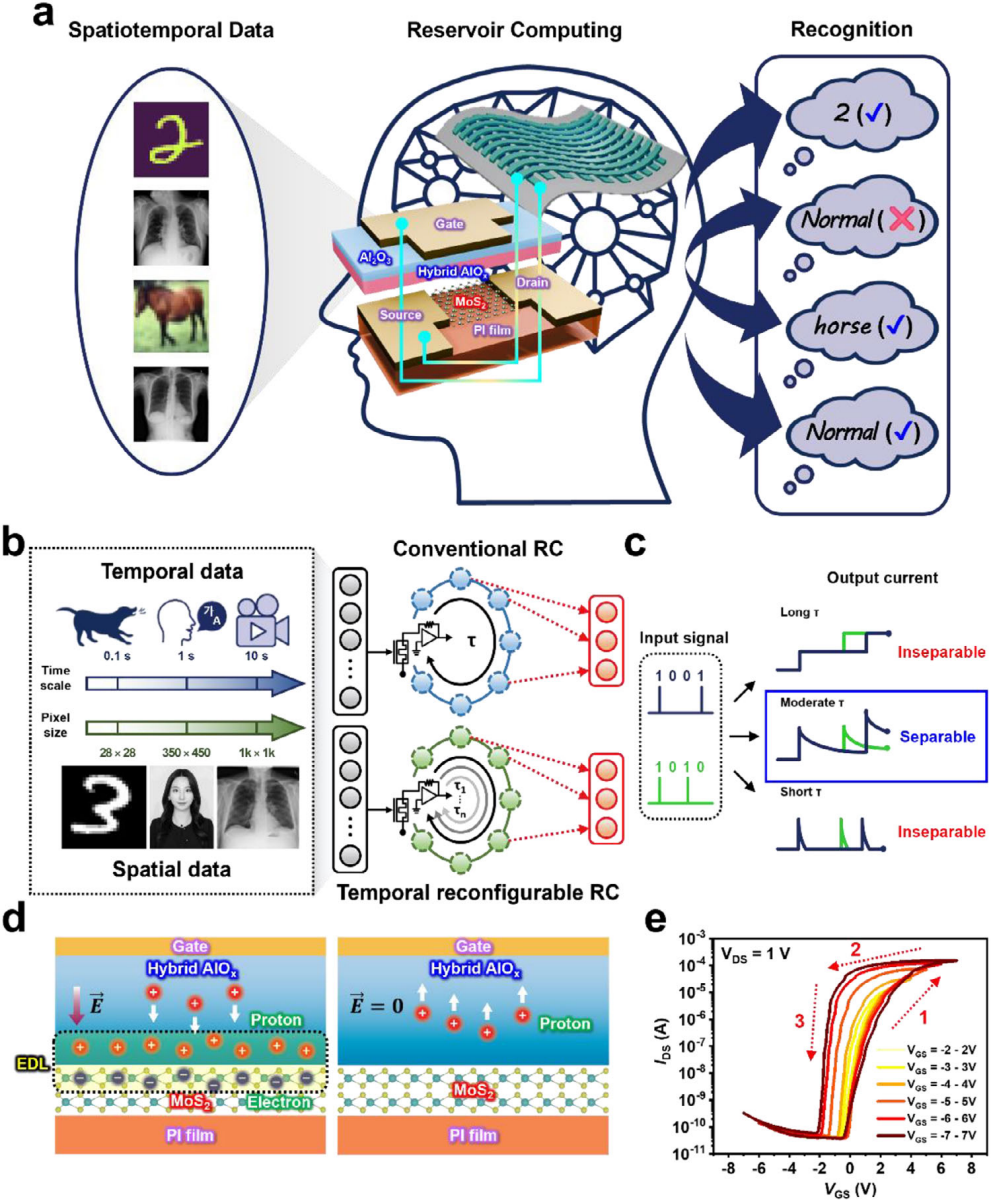
Temporally reconfigurable RC based on UFLEX TFT. a) Schematic of the concept of temporally reconfigurable RC based on UFLEX TFT. b) Structure of a conventional RC and a temporally reconfigurable RC. Real‐world spatiotemporal data is distributed across various timescales and spatial complexities. Temporally reconfigurable RC, with its multiple temporal dynamics, overcomes the limitations of conventional RC, which is constrained by fixed temporal dynamics, making it more advantageous for processing diverse temporal patterns. c) The temporal dynamics of the reservoir critically influence the separability of reservoir states. To optimize the RC system, an appropriate temporal scale is essential–neither too long nor too short. d) Schematic representation depicting proton movement within the hybrid AlO_x_ under positive bias and after bias removal. e) Transfer curve of the fabricated UFLEX TFT.

In this study, we demonstrate a temporally reconfigurable RC system using a simple electrical signal input method, enabling precise control over various temporal dynamics. To achieve this, we employ electrolyte‐gated transistors (EGTs), which have garnered attention as neuromorphic computing devices due to their simple device structure, low‐power operation, and excellent endurance. To overcome the scalability and gate controllability limitations of conventional EGTs that utilize liquid electrolytes, we develop ultrathin, flexible, all‐solid‐state electrolyte‐gated thin‐film transistors (UFLEX TFTs; **Figure** **1**a) and utilize them as physical reservoir devices.

Electrolyte‐based transistors incorporating protons form an ultrathin electric double layer (EDL) at the electrolyte/channel interface, with a thickness of ≈1 nm. This results in an exceptionally high capacitance (≈1–10 µF cm^−2^), which significantly outperforms conventional field‐effect transistors (FETs),^[^ ^14^ ^]^ thereby ensuring superior gate‐channel coupling efficiency.^[^ ^15^ ^]^ Furthermore, to enhance gate modulation efficiency and support EDL formation, we adopt molybdenum disulfide (MoS_2_), a representative material of 2D transition metal dichalcogenides (TMDCs), as the transistor channel. MoS_2_ not only exhibits high electron density at atomic thicknesses but also benefits from its atomically thin nature, which further contributes to efficient gate modulation.^[^ ^16^ ^]^ By integrating MoS_2_ as the channel material and employing organic–inorganic hybrid aluminum oxide (AlO_x_) synthesized via initiated chemical vapor deposition (iCVD) as both the electrolyte and gate dielectric, UFLEX TFTs exhibit remarkable electrical properties. These include a high on/off current ratio (≈10^7^), excellent endurance (2.5 × 10^4^ pulses), outstanding cycle‐to‐cycle uniformity (mean coefficient of variation of 0.091), device‐to‐device uniformity, and a wide memory window.

The proposed device is fabricated on a flexible polyimide (PI) substrate, leveraging the inherent flexibility of MoS_2_ and hybrid AlO_x_. Combined with its low‐power operation—a key characteristic of neuromorphic processors—UFLEX TFTs are well‐suited for flexible edge devices. The device maintains high accuracy even after undergoing the bending strain tests and the cyclic bending tests, demonstrating its potential for high‐performance flexible RC hardware.

Through efficient tuning of temporal dynamics, UFLEX TFTs provide a rich reservoir space, achieving a device‐to‐system simulation accuracy of 93.7% in handwritten digit recognition using the Modified National Institute of Standards and Technology (MNIST) dataset. Furthermore, we demonstrate the applicability of our system to object recognition using the Canadian Institute for Advanced Research (CIFAR) dataset, achieving an accuracy of 90.3% in classifying real‐world color object images. Leveraging the high computational performance of the proposed RC system, we also successfully perform chest X‐ray image classification—traditionally processed using convolutional neural networks (CNNs)—via RC for the first time, attaining an accuracy of 81.8%.

This study presents UFLEX TFT‐based temporally reconfigurable RC as a promising alternative to conventional machine learning approaches, addressing the high computational cost associated with complex clinical data analysis. Additionally, by adopting a low‐temperature back‐end‐of‐line (BEOL) compatible fabrication process, we realize a flexible and structurally simple device, paving the way for high‐performance, energy‐efficient flexible neuromorphic computing applications.

## 2 Results and Discussion

### 2.1 Temporally Reconfigurable Reservoir Computing with UFLEX TFTs

Figure 1b illustrates the structure of the RC systems, which comprises multiple dynamic nodes and a readout network implemented on a single physical device. This device functions as a reservoir, where the current response varies based on the input stimulus and STM. Due to the distinct temporal characteristics of each input, unique current outputs are generated. The device conductivity serves as a valid input for the readout network, enabling the classification of temporal input sequences.

However, for accurate classification, the input interval must align with the device's STM timescale (τ). If τ is too long, all inputs equally influence the final output, causing the effects of previous inputs to blur. Conversely, if τ is too short, prior inputs decay too rapidly and fail to integrate with subsequent inputs. Figure 1c illustrates this phenomenon, where reservoir output increases due to input spikes and then decays due to STM effects. When τ is excessively long or short, the system confuses similar input patterns (e.g., “1001” and “1010”), leading to classification errors. Therefore, only an optimal τ can generate distinguishable reservoir states. Since real‐world input data are distributed across various timescales, we propose a temporally reconfigurable reservoir structure capable of adjusting temporal dynamics optimized for each input. In this structure, a device with tunable STM properties plays a crucial role.

The STM of the UFLEX TFT in this study arises from the formation and dissipation of an EDL induced by cation migration within the electrolyte. Figure 1d presents a schematic representation of the switching mechanism of the EDL in the UFLEX TFT. When a positive gate voltage is applied, cations within the electrolyte migrate toward the channel, forming a positively charged layer. This layer induces electron accumulation in the MoS_2_ channel, forming the EDL. When the positive gate voltage is removed, the cations move away from the channel, leading to EDL dissipation.

Figure 1e presents the transfer characteristics of the UFLEX TFT, measured under vacuum (<10^−2^ Torr) condition with a dual sweep of gate voltage (*V*_GS_) from −7 to 7 V and a drain voltage (*V*_DS_) of 1 V. The device exhibits counterclockwise hysteresis, characterized by a higher threshold voltage (*V*_th_) during the forward sweep and a lower *V*_th_ during the backward sweep. The hysteresis window expands with increasing gate voltage range, a typical characteristic of memory devices. Utilizing a solid electrolyte with excellent insulating properties, this device demonstrates a high on/off ratio (≈10^7^) due to low gate leakage current and the absence of solution‐induced channel doping, which is commonly observed in liquid electrolyte‐based transistors. In the graph, three distinct processes are observed: 1) when a positive voltage is applied, cations accumulate and rearrange near the channel, gradually increasing *I*_DS_; 2) as the positive voltage is reduced, the cations near the channel decrease slightly; and 3) when a negative voltage is applied, the EDL rapidly collapses. This behavior is characteristic of the transfer curve of EGTs exhibiting typical EDL phenomena, demonstrating that the formation and dissipation rate of the EDL determines the relaxation time of STM properties.

### 2.2 Material Characterization of UFLEX TFT

**Figure 2:**
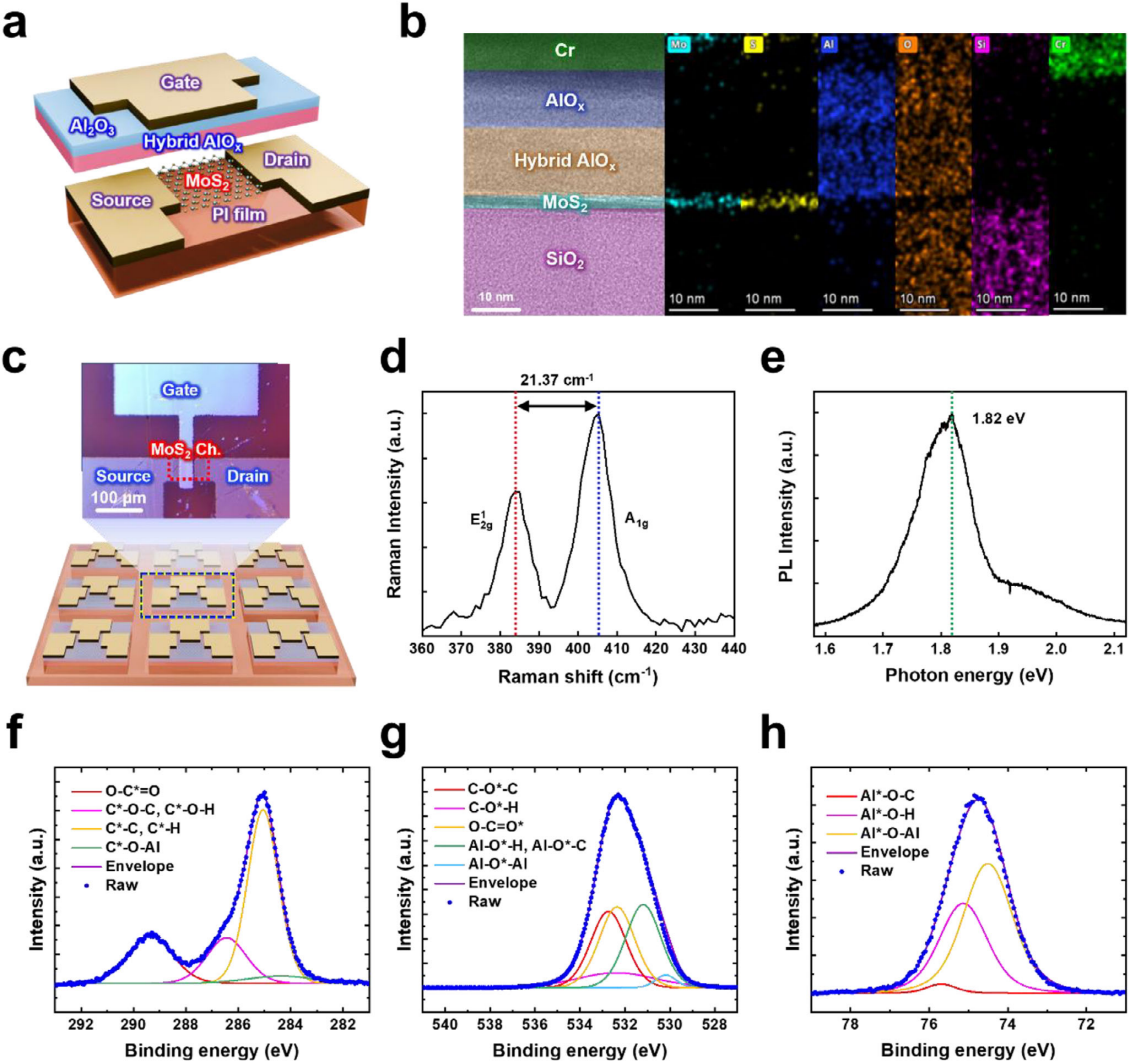
Design of the UFLEX TFT. a) Description of the 3D image of the fabricated UFLEX TFT. b) Cross‐sectional transmission electron microscopy (TEM) analysis of the UFLEX TFT. The left panel presents a high‐resolution TEM image of the UFLEX TFT, while the right panel displays energy dispersive X‐ray spectroscopy (EDS) mapping of Mo‐L, S‐K, O‐K, and Al‐K at the interface between the MoS_2_ channel and the electrolyte. c) A schematic illustration of the large area demonstrated UFLEX TFT, along with an optical microscope image of the fabricated device. The scale bar represents 100 µm. d) Raman spectrum of the synthesized MoS_2_ film. e) Photoluminescence (PL) spectrum of the synthesized MoS_2_ film. XPS spectra of f) the C 1s peak, g) the O 1s peak, and h) the Al 2p peak of the hybrid AlO_x_.

The proposed UFLEX TFT structure is illustrated in **Figure** **2**a. The gate, source, and drain electrodes consist of chromium (Cr) and gold (Au), deposited at thicknesses of 5  and 35 nm, respectively. The channel material comprises two layers of MoS_2_. To induce memory effects via the EDL, a 10 nm organic–inorganic hybrid AlO_x_ interlayer is incorporated as the electrolyte layer using initiated chemical vapor deposition (iCVD) at a stage temperature of 40 °C, and a 12 nm Al_2_O_3_ gate dielectric is deposited via atomic layer deposition (ALD) at 150 °C. The hybrid AlO_x_ is synthesized by polymerizing trimethylaluminum (TMA) within a 2‐hydroxyethyl methacrylate (HEMA) matrix, which serves as the structural backbone (Figure S1, Supporting Information). The hydroxyl groups in HEMA react with TMA to form an AlO_x_‐grafted hybrid insulator, where protons bonded to aluminum (Al) serve as mobile ions. A solvent‐free iCVD process is employed to fabricate the Al‐based solid electrolyte.

The iCVD technique operates by vaporizing initiators and monomers, generating radicals via heated filaments, and subsequently facilitating free radical polymerization to form polymer thin films.^[^ ^17^ ^]^ Due to its solvent‐free nature, iCVD enables the polymerization of functional copolymers, offering the advantage of forming nanometer‐thick, uniform metal‐organic hybrid copolymer films on a wafer scale.^[^ ^18^ ^]^

A cross‐sectional transmission electron microscopy (TEM) image of the UFLEX TFT is shown in Figure 2b, revealing a well‐defined interface between the channel and gate dielectric. Figure 2c presents an optical microscope image of the MoS_2_/hybrid AlO_x_‐based UFLEX TFT, where the top‐gated transistor structure is demonstrated using photolithography. The channel width and length are 40 and 10 µm, respectively.

Raman spectroscopy was employed to analyze the MoS_2_ synthesized via chemical vapor deposition (CVD). As shown in Figure 2d, the A_1g_ (405.25 cm^−1^) and E^1^_2g_ (383.88 cm^−1^) peaks exhibit a narrow frequency difference of Δk = 21.37 cm^−1^, confirming the formation of bilayer MoS_2_.^[^ ^19^ ^]^ This ultrathin MoS_2_ exhibits excellent gate controllability, high electron mobility, and a high on/off current ratio, making it a promising channel material for high‐performance all‐solid‐state electrolyte‐gated TFTs. Additionally, Figure S2 (Supporting Information) presents an optical image of the triangular MoS_2_ thin film, and the photoluminescence (PL) analysis (Figure 2e) provides further evidence that the synthesized MoS_2_ consists of both monolayer and bilayer regions, with a peak at 1.82 eV.^[^ ^19^, ^20^ ^]^

Conventional EGTs typically employ liquid electrolytes, which lead to high leakage currents and power consumption while limiting the device architecture to bottom‐gate configurations due to challenges in thickness scalability. In contrast, our UFLEX TFT leverages an iCVD‐polymerized solid electrolyte, enabling a top‐gate structure that significantly enhances device integration density. Furthermore, its superior scalability, excellent gate controllability, and reduced power consumption facilitate the fabrication of ultrathin TFTs. The development of atomic‐scale thin channels and nanometer‐scale solid electrolytes is thus crucial for improving device performance.

The movement of protons within hybrid AlO_x_ follows the Grotthuss mechanism, wherein protons undergo rapid and continuous hopping along a hydrogen bonding network through the dynamic transformation of O─H covalent bonds and O···H─O hydrogen bonds within the polymer matrix.^[^ ^21^ ^]^ Therefore, to assess the proton storage capability of UFLEX TFTs and the extent of dynamic responses facilitated by proton hopping, it is essential to evaluate the density of hydrogen bonding networks within the electrolyte layer. X‐ray photoelectron spectroscopy (XPS) was employed to analyze the atomic composition and chemical bonding states of the synthesized hybrid AlO_x_.

Figure 2f presents the deconvolution spectra of the C 1s peak derived from XPS analysis. While the C─O─H bond involves a hydrogen atom, its strong covalent nature precludes its participation in the hydrogen bonding network relevant to proton hopping. Figure 2g shows the deconvolution spectra of O 1s, where the Al─O*─H bond, which is involved in the hydrogen bonding network, exhibits a peak at 531.2 eV, accounting for 30.3% of the total O 1s signal. Figure 2h illustrates the deconvolution spectra of Al 2p, where the Al*─O─H bond, associated with the hydrogen bonding network, is located at 75.2 eV and comprises 38.9% of the total Al 2p signal. These results indicate that the hybrid AlO_x_ electrolyte layer possesses substantial proton storage capacity and enables dynamic proton hopping.^[^ ^18^, ^22^ ^]^

Figure S3 (Supporting Information) demonstrates the enhancement in capacitance with temperature variation. It can be observed that at 50 Hz, the capacitance of the hybrid AlO_x_ film increases by 19.2 and 10.4% at 300 and 372 K, respectively, compared to its value at 100 kHz. This improvement primarily originates from the polarization of the insulator within the film and proton polarization induced by low‐frequency AC voltage, confirming the presence of a substantial amount of protons.^[^ ^23^ ^]^ Consequently, the abundant protons in UFLEX TFTs contribute to the formation of EDL under positive bias, thereby enhancing the superior gate control characteristics of EGTs.

### 2.3 Modulation of Temporal Dynamics and Reliability Assessment

To investigate the modulation mechanism of STM, we measure and analyze the drain current response under various input voltage pulses. Figure S4 (Supporting Information) illustrates the voltage waveforms applied to the gate electrode during the measurements. Three pulse parameters were considered: pulse duration (*t*_pulse_); pulse magnitude (*V*_mag_); and background voltage (*V*_bg_). Throughout the experiment, a constant voltage of 1 V was maintained at the drain electrode. Figure S5 (Supporting Information) presents the drain current response to a single positive gate voltage pulse. Upon pulse application, the drain current (*I*_DS_) exhibits a sharp increase (Δ*I*_DS_), followed by a gradual relaxation, eventually returning to its initial state. This STM behavior aligns well with the stretched exponential model, expressed as follows:

(1) $$I\left(t\right)=I_{0}+\Delta I_{DS}\exp\left[-{\left(\frac{t-t_{0}}{\tau }\right)}^{\beta }\right]$$

This model is widely employed to characterize the relaxation mechanism in systems containing randomly distributed traps.^[^ ^24^ ^]^ It incorporates a suppression factor, β ∈ (0,1), which modifies the classical exponential decay by accounting for the decreasing recombination rate as traps become depleted. The consistency between the experimental data and this model supports the underlying physical mechanism.

**Figure 3:**
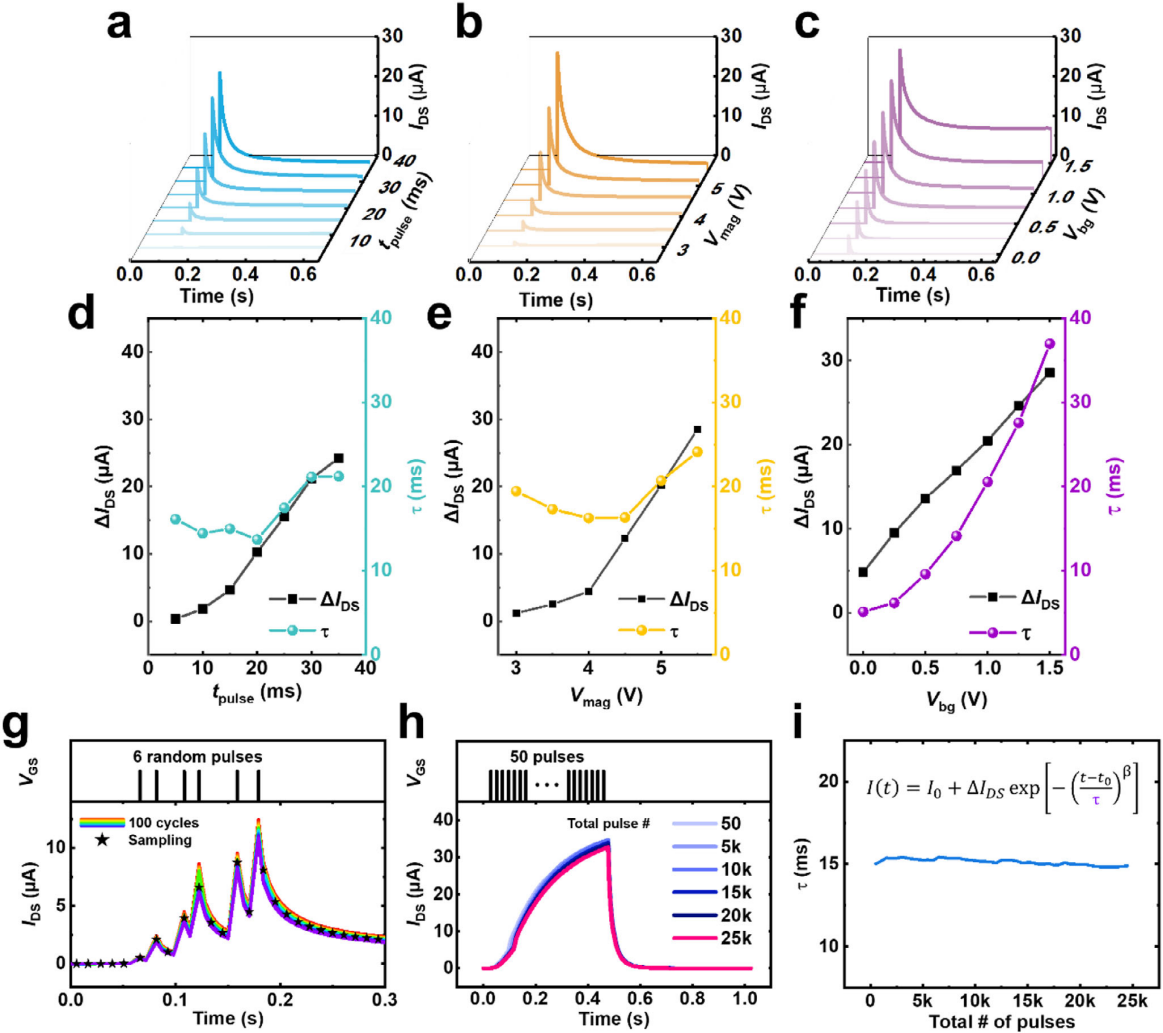
STM modulation and reliability characteristics. a,d) STM behavior under varying pulse durations (5–35 ms) at *V*_mag_ = 5 V, *V*_bg_ = 1 V, and *V*_DS_ = 1 V. Increasing the pulse duration enhances Δ*I*_DS_ while having a negligible effect on τ. b,e) STM behavior under different *V*_mag_ values (3.5–5.5 V) with a fixed pulse duration of 30 ms, *V*_bg_ = 1 V, and *V*_DS_ = 1 V. *V*_mag_ facilitates Δ*I*_DS_ without significantly affecting τ. c,f) STM behavior under varying *V*_bg_ values (0–1.5 V) at a pulse duration of 30 ms, *V*_mag_ = 5 V, and *V*_DS_ = 1 V. *V*_bg_ enhances both Δ*I*_DS_ and τ, leading to a substantial modulation of the relaxation time. g) Cycle‐to‐cycle variation test of the UFLEX TFT. The identical spike sequence is repeatedly applied to the gate electrode 100 times, exhibiting stable readout current uniformity. h) Endurance test of the UFLEX TFT. One cycle consists of 50 consecutive pulses (*V*_mag_ = 5 V, 5 ms width and interval, *V*_DS_ = 1 V, *V*_bg_ = 0 V), and the cycle is repeated 500 times, resulting in a total of 2.5 × 10⁴ pulses. The current rapidly decreases after the last pulse in each cycle due to the short pulse duration and lack of background bias, confirming stable STM behavior even after the full 2.5 × 10⁴ pulses. i) Evolution of τ during the endurance test. The stability of τ remains consistent over 2.5 × 10^4^ pulses.

Subsequently, we investigate the effect of input pulse parameters on the response current (Δ*I*_DS_) and the relaxation time constant (τ). **Figure** **3**a presents the current response for various pulse durations (5–35 ms). As the *t*_pulse_ increases, Δ*I*_DS_ becomes larger, while τ remains nearly unchanged (Figure 3d). This behavior arises because the redistribution of electrons and the subsequent increase in current are more pronounced with longer activation time. Figure 3b presents the current response for various pulse magnitudes (*V*_mag_, 3.0–5.5 V). Likewise, higher *V*_mag_ increase Δ*I*_DS_ without significantly affecting τ (Figure 3e). This result suggests that, while a stronger bias accelerates the activation process, the relaxation process remains independent of activation conditions. We further investigate the drain current response under different background voltage conditions (0–1.5 V), as shown in Figure 3C,F. Both Δ*I*_DS_ and τ exhibit a strong dependence on *V*_bg_. Notably, as *V*_bg_ increases, τ becomes larger, indicating a slower relaxation process. The modulation range of τ induced by *V*_bg_ consistently expands from ≈11 to 191 ms (Figure S6, Supporting Information), suggesting that a broader *V*_bg_ range enables greater modulation flexibility. This behavior may be attributed to the presence of a background positive gate voltage even after the pulse application, which slows the relaxation process. The sustained localization of protons near the channel after gate voltage pulse application maintains the EDL, thereby delaying dissipation. A schematic illustration of how the *V*_bg_ modulates proton relaxation dynamics and short‐term memory characteristics is provided in Figure S7 (Supporting Information).

Finally, the impact of *V*_bg_ modulation on various device parameters is investigated. The results indicate that while β remains unaffected, the initial current increases with higher *V*_bg_ values (Figure S8, Supporting Information). The impact of *V*_bg_ on the initial current state is further analyzed in Figure S9 (Supporting Information). In summary, the temporal dynamics of UFLEX TFTs can be effectively tuned over a wide range, facilitating the implementation of temporally reconfigurable RC hardware platforms.

Since the cycle‐to‐cycle uniformity of the hardware reservoir is a critical metric that directly impacts RC performance, it is evaluated by repeatedly applying a specific pulse sequence 100 times and analyzing the resulting variations (Figure 3g). Specifically, a sequence of six gate pulses with randomly assigned timing, while maintaining identical amplitude and duration, was applied to the device and repeated for 100 cycles. The results reveal highly consistent STM behavior across cycles, with nearly identical τ and minimal Δ*I*_DS_ variations. To further quantify this uniformity, we analyze 25 uniformly sampled current values per cycle, yielding a mean σ/µ of 0.091 (Figure S10, Supporting Information). Additionally, transfer curves obtained from *V*_GS_ dual sweep measurements on 20 randomly selected devices exhibit consistent hysteresis behavior (Figure S11, Supporting Information). To evaluate how device variation affects system‐level RC performance, we conducted classification simulations incorporating both cycle‐to‐cycle and device‐to‐device variation (Figure S12, Supporting Information). RC simulations were performed using the drain current outputs obtained from 20 repeated pulse stimulations (cycle‐to‐cycle) and 20 randomly selected devices (device‐to‐device) under five different conditions: *V*_bg_ = 0, 200, and 400 mV, bending strain (R = 3.5 mm), and cyclic bending (1000 cycles). These current sequences were used to classify the MNIST handwritten digit, CIFAR‐10 object image, and NIH ChestX‐ray image datasets. As summarized in Figure S12 (Supporting Information), the resulting accuracy distributions across all conditions and datasets exhibit small standard deviations, validating that the low device‐level variability of UFLEX TFTs translates into high consistency in system‐level RC performance.

To assess the endurance of the hardware reservoir, we apply one cycle consisting of 50 consecutive positive pulses (*V*_mag_ = 5 V, *t*_pulse_ = 5 ms, inter‐pulse interval = 5 ms, *V*_DS_ = 1 V, and *V*_bg_ = 0 V) to the gate electrode, and repeat this cycle 500 times, resulting in a total of 2.5 × 10^4^ pulses and analyze the resulting STM behavior (Figure 3h). In the Figure 3h, the legends “50,” “5k,” and “25k” correspond to the drain current responses measured after the 50th pulse (end of the 1st cycle), after the 5000th pulse (end of the 100th cycle), and after the 25 000th pulse (end of the 500th cycle), respectively. During the pulse train, the drain current gradually increases due to transient proton accumulation and then rapidly decreases after the final pulse. This sharp decay, caused by the short pulse duration and the absence of a background gate bias, reflects the volatile dynamics of the reservoir and aligns with the short‐term memory characteristics required for temporal processing. Even after 2.5 × 10^4^ pulses, the response remains stable with minimal drift, indicating reliable STM operation. The sustained stability of *I*_DS_, even after an extensive number of input pulses, is attributed to the structural and material robustness of the UFLEX TFT. The use of CVD‐grown MoS_2_ as the channel material ensures high crystalline quality and mechanical integrity, which are essential for maintaining consistent electronic transport characteristics during repeated electrical stimulation. In addition, the incorporation of a solid‐state hybrid AlO_x_ electrolyte, deposited via iCVD, enhances electrochemical stability and suppresses ionic degradation mechanisms commonly observed in liquid electrolyte‐based transistors. These material choices collectively contribute to the reliable preservation of short‐term memory behavior under prolonged pulsing conditions. It is important to clarify that the STM behavior observed in our devices is highly relevant to RC. Unlike synaptic devices, which benefit from persistent potentiation and depression, reservoirs rely on fast relaxation of internal states to retain recent inputs without interference from long‐past stimuli. In this context, the sharp decay of the drain current following a sequence of pulses reflects the rapid dissipation of the EDL, which is a desirable characteristic for STM operation. Furthermore, Figure 3i confirms that τ exhibits negligible performance degradation even after 2.5 × 10^4^ pulses. This exceptional endurance beyond 2.5 × 10^4^ pulses underscores the robustness of the physical reservoir, making it well‐suited for hardware‐based RC system implementation.

All data in Figure 3 were obtained from devices fabricated under identical process conditions. The observed variation is due to differences in the stimulation protocols—isolated or spaced pulses vs continuous sequences—and not due to intrinsic differences in device behavior. These results confirm that our devices maintain robust STM characteristics across different conditions, supporting their suitability as physical reservoirs.

### 2.4 Temporal Reconfigurability and Mechanical Flexibility

**Figure 4:**
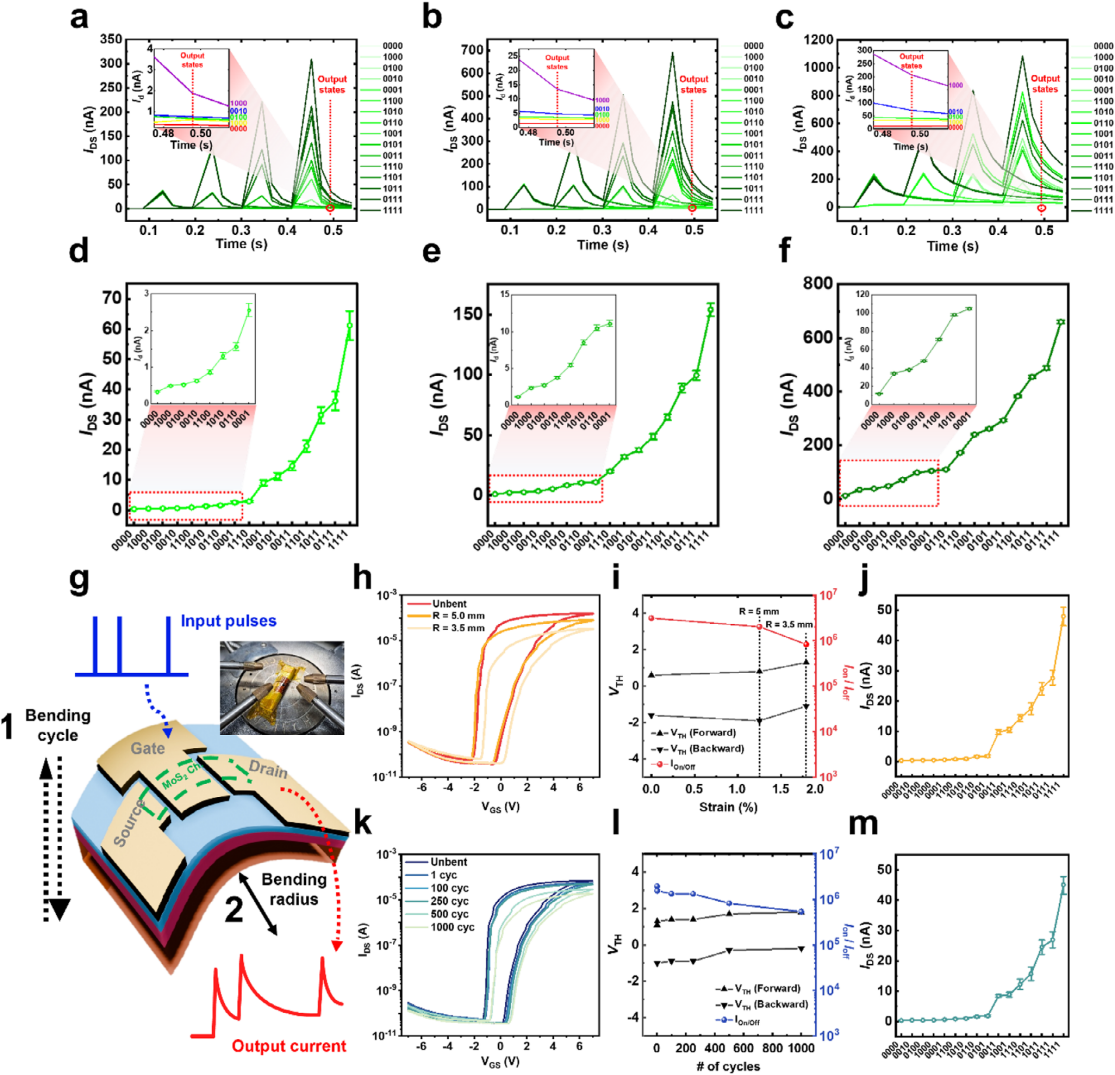
Demonstration of separability and mechanical flexibility. a–c) Experimental readout currents of the UFLEX TFT for 16 different 4‐bit pulse sequences at a) *V*_bg_ = 0 mV, b) *V*_bg_ = 200 mV, and c) *V*_bg_ = 400 mV. d–f) Reservoir state separability outputs for 16 different 4‐bit pulse sequences at d) *V*_bg_ = 0 mV, e) *V*_bg_ = 200 mV, and f) *V*_bg_ = 400 mV. Insets in a–f) show magnified views of the output current values corresponding to pulse sequences ranging from 1000 to 0001. g) Schematic of bending tests: bending strain test and cyclic bending test. h,k) Transfer curves of the UFLEX TFT: h) after the bending strain test, k) after the cyclic bending test. i,l) Quantitative comparison of the threshold voltages and the on/off current ratio of the UFLEX TFT under varying i) strain and l) bending cycle counts. j,m) Reservoir state separability outputs for 16 different 4‐bit pulse sequences: j) after the bending strain test, m) after the cyclic bending test.

To develop a hardware RC system utilizing UFLEX TFTs, we evaluate the fundamental characteristic of state separability. A series of 4‐bit pulse sequences, representing 16 unique inputs in the range of 0000–1111, is applied under three different *V*_bg_ conditions (0, 200 , and 400 mV), while maintaining a fixed *V*_DS_ of 1 V (**Figure** **4**a–c). The reservoir states are determined by sampling the output current, obtained from 30 devices, after each pulse sequence is applied (Figure 4d–f). Under all *V*_bg_ conditions, the results for the 16 pulse sequences effectively minimize state overlap, highlighting the device's excellent state separability. To more clearly demonstrate the distinguishability of output states among similar pulse sequences—including those ranging from 0000 to 0001—magnified inset plots were incorporated in Figure 4a—f. These insets reveal that the UFLEX TFT produces distinct output currents even for sequences with subtle differences, thereby supporting reliable state separability under low *V*_bg_ conditions.

For edge computing applications, achieving low‐power operation is crucial, while mechanical flexibility is a key advantage for flexible electronics. To further validate low‐power operation, we quantified the instantaneous power consumption of the UFLEX TFT during RC operation under a read voltage of 1 V. As shown in Figure 4a–c, the drain current ranges from ≈31.5 nA (*V*_bg_ = 0 mV, input sequence: 1000, first peak) to 1.08 µA (*V*_bg_ = 400 mV, input sequence: 1111, last peak), corresponding to a power consumption range of 31.5 nW to 1.08 µW. This low power consumption is significantly lower than those reported for other neuromorphic devices such as ferroelectric FETs (≈60 µW–3 mW),^[^ ^30^ ^]^ ferroelectric tunneling junctions (≈70 µW),^[^ ^10^ ^]^ memristor‐based systems (≈1.5 µW–37.5 mW),^[^ ^8^, ^9^, ^31^ ^]^ and FPGAs (≈34 mW–1.36 W).^[^ ^32^ ^]^ A benchmark comparison of power and accuracy is presented in Figure S13 (Supporting Information), demonstrating the unique combination of low power and high classification performance achieved by the UFLEX TFT. To evaluate flexibility, we analyze the electrical performance and separability through two types of tests: bending strain tests and cyclic bending tests (Figure 4g). The strain induced in the device is calculated using the equation ɛ = 𝑡/(2𝑅),^[^ ^25^ ^]^ where 𝑡 represents the device thickness and 𝑅 denotes the bending radius. Since the thickness of the PI substrate (125 µm) is significantly larger than the total device layer thickness (80 nm), the strain induced in the device layers can be considered negligible and is assumed to follow that of the substrate.

Figure 4h presents the transfer curves of UFLEX TFTs measured in a vacuum chamber (< 10^−2^ Torr) under bent conditions with radii of 5.0 mm (ɛ = 1.25%) and 3.5 mm (ɛ = 1.79%), using a dual‐sweep *V*_GS_ ranging from −7 to 7 V and a *V*_DS_ of 1 V. The results indicate a slight rightward shift in the transfer curve and a minor reduction in on current (*I*_on_) at 𝑅 = 3.5 mm (ɛ = 1.79%), suggesting a marginal degradation in electrical performance. However, the transfer curves remain well‐defined, demonstrating stable transistor operation. To quantitatively assess performance degradation under bending, we extract the threshold voltages and the on/off current ratio as functions of strain (Figure 4i). While the on/off current ratio gradually decreases as R is reduced to 3.5 mm, it remains close to 10^6^. The hysteresis window increases slightly from 2.2  to 2.4 V, confirming stable and reliable *I*_DS_–*V*_GS_ transfer characteristics under bending. After the bending strain tests, we evaluate the state separability of the reservoir using 4‐bit pulse input sequences under *V*_bg_ = 0 mV, obtained from 10 devices (Figure 4j). Although *I*_DS_ values show a slight reduction compared to flat‐state conditions, the physical reservoir maintains clear state separability.

For cyclic bending tests, we apply the same vacuum chamber conditions, gate voltage sweeps, and drain voltage settings. As shown in Figure 4k, the transfer curves exhibit a slight rightward shift and a minor reduction in *I*_on_ over 1000 bending cycles under 1.25% strain, yet the device retains stable operation. Figure 4l quantitatively illustrates the degradation of the forward and reverse threshold voltages and the on/off current ratio as functions of the bending cycle count. The on/off ratio decreases slightly but remains above 5 × 10^5^, while the hysteresis window decreases slightly from 2.1  to 2.0 V, confirming stable *I*_DS_–*V*_GS_ characteristics under repeated bending. After cyclic bending, we assess the reservoir state separability using the same 4‐bit pulse sequence method, obtained from 10 devices (Figure 4m). The overall *I*_DS_ values show a slight reduction compared to flat state conditions. Compared to the bending strain test results, we observe an increased overlap in the output current values, particularly for the “1011” and “0111” sequences, indicating a minor degradation in separability under cyclic bending stress. However, the device continues to function reliably as a physical reservoir.

**Figure 5:**
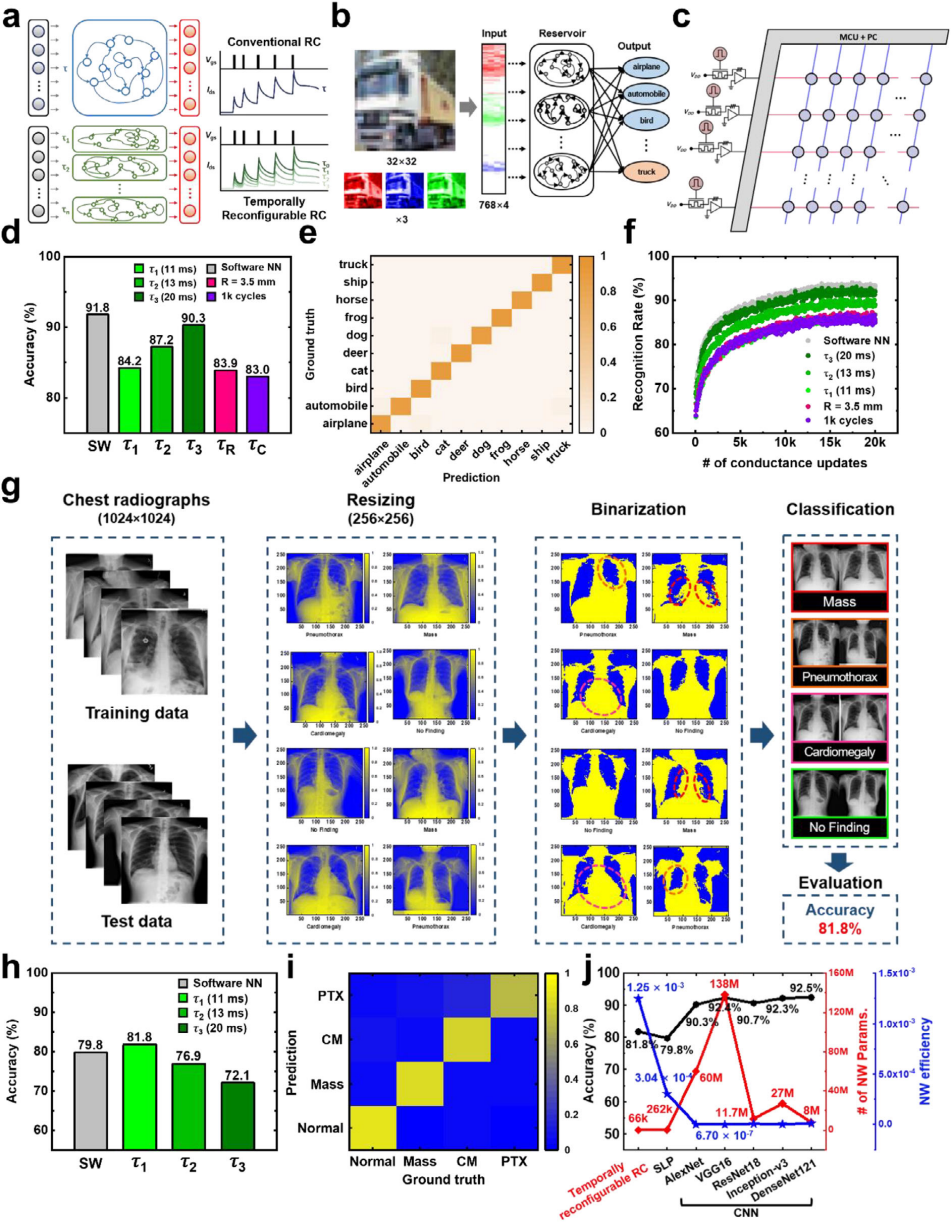
Device‐to‐system level simulations. a) Schematic comparison of the conventional RC and temporally reconfigurable RC architectures. b) Process flow of 32 × 32 pixel CIFAR‐10 images with three color channels classification. c) Schematic of the hardware‐based RC system implemented with UFLEX TFTs. d) Comparison of experimental accuracies on the CIFAR‐10 dataset across different temporal characteristics. As τ increases (τ_1_ = 11 ms, τ_2_ = 13 ms, τ_3_ = 20 ms), accuracy improves. The performance degradation of UFLEX TFTs after bending tests is negligible. e) Confusion matrix of the experimental recognition results on the CIFAR‐10 dataset under the τ_3_ condition. f) Training performance of the hardware‐implemented temporally reconfigurable RC system with UFLEX TFTs on the CIFAR‐10 dataset. g) Workflow of the RC system processing for NIH chest X‐ray image classification. h) Comparison of experimental accuracy on the NIH chest X‐ray across different temporal characteristics. As τ decreases, accuracy improves. i) Confusion matrix of the experimental recognition results on the NIH chest X‐ray dataset under the τ_1_ condition. j) Performance comparison of the temporally reconfigurable RC system and various software‐based neural networks on the NIH chest X‐ray dataset.

The UFLEX TFT, utilizing an ultrathin MoS_2_ layer, effectively prevents buckling commonly observed in thicker exfoliated MoS_2_ films, thereby maintaining high drive currents even under bending conditions.^[^ ^26^ ^]^ Furthermore, instead of relying solely on conventional inorganic Al_2_O_3_, the device employs an Al‐containing hybrid electrolyte dielectric, achieving excellent structural stability and performance retention under bending stress. This enhancement arises from the combination of the flexibility of organic components and the electrical stability of inorganic components, effectively improving resistance to mechanical deformation. Finally, we apply the UFLEX TFTs subjected to two types of bending tests to device‐to‐system‐level simulations for classification tasks using both the MNIST handwritten digit dataset and the CIFAR‐10 color image dataset. Our UFLEX TFT‐based RC system achieves classification accuracies of 92.0 and 91.8% for MNIST, and 83.9 and 83.0% for CIFAR‐10 under the bending strain and cyclic bending tests, respectively (**Figure** **5**d; Figure S14, Supporting Information). These results demonstrate that UFLEX TFTs not only exhibit excellent electrical performance but also maintain robust neuromorphic functionality under mechanical stress, confirming their applicability to wearable reservoir devices for edge computing.

A comprehensive benchmarking comparison with other representative electrolyte‐gated TFTs is summarized in Table S1 (Supporting Information), clearly demonstrating the superior mechanical flexibility, robustness over 1000 bending cycles, and high classification accuracies of our device, thereby underscoring its competitiveness for flexible neuromorphic computing applications.

### 2.5 Device‐to‐System Level Simulations

The manufactured UFLEX TFT facilitates hardware‐implemented temporally reconfigurable RC system. As shown in Figure S15 (Supporting Information), conventional neural networks consist of a linear synaptic layer interspersed between nonlinear neuron layers, where all synaptic weights participate in training. In contrast, as shown in the upper part of Figure 5a, RC employs fixed reservoir nodes to project input signals into a high dimensional computational space, only the trainable output synaptic layer participates in training. This approach, which leverages linear regression for training, enables efficient learning with minimal computational cost. However, simplifying the network may trade off computational performance.

To overcome this limitation, incorporating sub‐reservoirs with diverse temporal dynamics, as illustrated in the lower part of Figure 5a, allows for the processing of temporal signals over varying timescales. In hardware‐based reservoirs, these temporal dynamics are inherently governed by the properties of the materials and the fabrication processes. However, the proposed UFLEX TFT integrates an input scheme incorporating *V*_bg_ modulation, allowing for tunable temporal dynamics (Tables S2, S3, Supporting Information). In contrast to previous approaches requiring external optical control or sophisticated pulse engineering, the UFLEX TFT enables direct and real‐time modulation of relaxation time using a single background gate voltage applied to the top gate. This simplicity not only facilitates compact device integration but also minimizes circuit‐level complexity for temporal reconfigurability.

Although Figure 1a illustrates a conceptual UFLEX TFT array for hardware RC applications, our experimental implementation and system‐level simulation were based on the measured responses of individual fabricated devices. Specifically, all 16 possible 4‐bit input pulse sequences (from “0000” to “1111”) were applied to UFLEX TFTs, and the corresponding output currents were recorded. These measured values were stored as a fixed mapping between input sequences and reservoir states, and were applied to each input during the subsequent simulation. This approach enabled a device‐to‐system‐level evaluation of RC performance without the need to fabricate a full transistor array.

Figures 5b and S16, Supporting Information illustrate the transformation of pixel intensities in the 32 × 32 pixel CIFAR‐10 images with 3 color channels (RGB) and the 28 × 28 pixel MNIST images into 4‐bit digital input pulse trains, generating a 768 × 4 pixel CIFAR‐10 dataset and a 196 × 4 pixel MNIST dataset, respectively, for performance evaluation. Figure 5c presents a schematic representation of the hardware‐based RC system with temporal reconfigurability, implemented using UFLEX TFTs. Reservoir nodes are linearly connected within a hardware‐implemented artificial synapse crossbar array or a CMOS array functioning as the readout layer.

Figure 5d compares the inference accuracy of a software‐based neural network consisting of 3072 × 10 neurons with that of an RC network employing 768 × 10 reservoir nodes based on UFLEX TFT. The RC network is evaluated under three *V*_bg_ conditions (τ_1_: *V*_bg_ = 0 mV, τ_2_: *V*_bg_ = 200 mV, and τ_3_: *V*_bg_ = 400 mV), as well as under two types of bending conditions: the bending strain test and the cyclic bending test. Using 50 000 training images, the software‐based neural network achieves a recognition accuracy of 91.8%. The hardware RC network based on UFLEX TFT exhibits improved recognition accuracy with increasing relaxation time, reaching a peak accuracy of 90.3% under the τ_3_ condition. Despite the software‐based network utilizing four times as many neuron nodes with higher computational complexity, the performance gap remains minimal. A similar trend is observed in the MNIST handwritten digit classification task, where the recognition accuracy of the UFLEX TFT‐based RC network improves with increasing relaxation time, approaching that of the software‐based neural network (Figure S14, Supporting Information). Unlike conventional RC systems, where each sub‐reservoir corresponds to a single RC device, the UFLEX TFT‐based temporally reconfigurable RC system assigns multiple sub‐reservoirs per device, optimizing performance under the most favorable sub‐reservoir conditions.

Furthermore, even after undergoing bending with a radius of 3.5 mm and 1000 cyclic bending cycles, the UFLEX TFT‐based RC system demonstrates exceptional reservoir state separability, with minimal performance degradation of only 0.3 and 1.2% compared to the τ_1_ condition (*V*_bg_ = 0 mV). The confusion matrix of the classification results under the τ_3_ condition is shown in Figure 5e. The confusion matrices for classification results under other conditions are presented in Figure S17 (Supporting Information). After 20 000 training iterations, all ten image classes (“airplane”, “automobile”, “bird”, “cat”, “deer”, “dog”, “frog”, “horse”, “ship”, and “truck”) are accurately classified for most test set samples, confirming the superior performance of this RC system. Additionally, the simulation results evaluating the training performance of the UFLEX TFT (Figure 5f) indicate that the recognition accuracy under the τ_3_ condition reaches 92.1%, exhibiting a difference of merely 1.2% compared to the software‐based neural network structure. In comparison, the MNIST handwritten digit classification task, performed after 20 000 training iterations, shows even higher accuracy in distinguishing all ten digit classes (0 through 9), achieving a recognition accuracy of 93.7% under the τ_3_ condition with only a 1.0% deviation from the software‐based neural network (Figures S14, S18, S19, Supporting Information).

Due to the exceptional temporal reconfigurability of the UFLEX TFT, this system can expand its applicability to more complex and higher‐resolution datasets beyond MNIST handwritten digit dataset. To validate this capability, the RC system is applied for the first time to classify and predict chest X‐ray images from the publicly available NIH ChestX‐ray14 dataset, which contains 1024 × 1024 pixel images across 15 classes. In this study, four classes– cardiomegaly, mass, pneumothorax, and no finding–are extracted from the NIH ChestX‐ray14 dataset. Each class consists of various images corresponding to the assigned labels.

The RC system processing workflow begins with data preprocessing, where each image is resized to 256 × 256 pixels, and the pixel intensity values are normalized between 0 and 1. Subsequently, the pixel intensities are converted into 4‐bit digital input pulse trains for processing (Figure 5g). The simulation employs a total of 7231 training data and 2769 inference data.

Figure 5h compares the inference accuracy of a software‐based neural network consisting of 65 536 × 4 neurons without reservoir nodes and a UFLEX TFT‐based hardware RC network comprising 16 384 × 4 neurons. As in the CIFAR‐10 simulation, the RC network performance is evaluated under three conditions: τ_1_ (*V*_bg_ = 0 mV); τ_2_ (*V*_bg_ = 200 mV); and τ_3_ (*V*_bg_ = 400 mV). Although the software‐based neural network achieves higher accuracy than the RC network in CIFAR‐10 object image classification, the RC network achieves a high inference accuracy of 81.8% under the τ_1_ condition in the NIH chest X‐ray classification task, surpassing the software‐based neural network by 2.0%.

The CIFAR‐10 dataset, consisting of 32 × 32 color object images, primarily contains simple patterns that are linearly separable. A single‐layer perceptron (SLP), which classifies input data through linear separation, can efficiently perform classification tasks without nonlinear transformations.^[^ ^27^ ^]^ Considering the relatively low resolution and simple structure of the CIFAR‐10 dataset, high classification performance can be achieved without complex nonlinear feature extraction. Consequently, an SLP provides high accuracy with minimal computational cost. In contrast, RC effectively models nonlinearity using complex dynamic systems,^[^ ^8^, ^28^ ^]^ however, such complexity is unnecessary for simple datasets such as CIFAR‐10. While an SLP exhibits rapid learning and high convergence efficiency for simple datasets,^[^ ^27^ ^]^ the intricate structure of RC may result in unnecessary computational complexity or even overfitting.

In contrast, chest X‐ray images exhibit significantly higher levels of nonlinearity and complexity compared to CIFAR‐10 images. These images contain intricate patterns and structures, such as lesion morphology, variations in tissue density, and shading effects, requiring advanced feature extraction techniques. Since RC processes input data in a high dimensional space by leveraging nonlinear dynamical systems, it is particularly well suited for capturing complex spatiotemporal patterns. For datasets such as chest X‐ray images, the RC system effectively learns and represents high dimensional complexity, demonstrating performance and efficiency that can surpass relatively simple software‐based neural networks such as SLP in recognizing intricate patterns.

Furthermore, the relationship between relaxation time (τ) and classification accuracy exhibits distinct trends in the CIFAR‐10 and chest X‐ray datasets. In CIFAR‐10 classification, accuracy increases with longer relaxation times (τ_1_ = 11 ms, τ_2_ = 13 ms, τ_3_ = 20 ms), whereas in NIH chest X‐ray classification, shorter relaxation times result in improved performance. This discrepancy reflects the interplay between the temporal dynamics of the reservoir and the characteristics of the input data. The CIFAR‐10 dataset consists of simple, spatially uniform patterns with a resolution of 32 × 32 pixels with 3 color channels. To classify digits, it is crucial to capture the overall shape, including geometric structures, curves, and edges. As shown in Figure 1c, a longer relaxation time enables the reservoir to retain information over extended periods, effectively preserving the global patterns of digits during computation. Consequently, the output layer can learn these global patterns more efficiently.^[^ ^8^, ^28^ ^]^ Therefore, for datasets such as CIFAR‐10, which exhibit low resolution and simple structures, longer relaxation times lead to improved classification performance.

In contrast, the chest X‐ray dataset relies on the detection of localized and highly detailed features. Accurate classification requires the ability to identify subtle differences within the images and leverage them effectively. A shorter relaxation time allows the reservoir to respond rapidly to recent inputs while efficiently discarding redundant past information, thereby improving the detection of instantaneous and localized features. As a result, in the NIH dataset, shorter relaxation times lead to enhanced classification performance. The confusion matrix illustrating the classification results of chest X‐ray images under τ_1_ ​condition is presented in Figure 5i. The confusion matrices depicting classification results under other conditions are presented in Figure S20 (Supporting Information). After 30 000 training epochs (Figure S21, Supporting Information), the majority of test samples are classified with high accuracy into four categories (normal, mass, cardiomegaly, and pneumothorax), demonstrating the high classification accuracy of the UFLEX TFT‐based temporally reconfigurable RC system.

Figure 5j compares the performance of the temporally reconfigurable RC system with that of software‐based artificial neural networks, including an SLP and various convolutional neural networks (CNNs), on the NIH chest X‐ray dataset. The CNN architectures under comparison—AlexNet, VGG16, ResNet18, Inception‐v3, and DenseNet121—are evaluated in terms of network parameters, classification accuracy, and network efficiency (N.E.). For CNN models, training is performed using 8500 training samples and 1344 inference samples, which is comparable to the dataset size used in this study, while being limited to two classification categories.^[^ ^29^ ^]^ To quantitatively compare model performance based on the number of network parameters, network efficiency (N.E.) is defined as follows:

(2) $$N.E.=\frac{\mathrm{Accuracy}}{\#\;\mathrm{of}\;\mathrm{network}\;\mathrm{parameters}}$$

Among the CNN models, the VGG16 architecture, which consists of 16 layers and utilizes 138 million network parameters, achieves an accuracy of 92.4%. Although VGG16 exhibits a substantial number of parameters and incurs a high computational cost, its performance difference from the temporally reconfigurable RC system remains relatively minor. In contrast, the temporally reconfigurable RC system employs only 65 536 network parameters while achieving an accuracy of 81.8%. When evaluated in terms of network efficiency, the RC system exhibits an N.E. value of 1.25 × 10^−3^ significantly outperforming VGG16, which records 6.70 × 10^−7^. This corresponds to a difference of over four orders of magnitude, highlighting the exceptional efficiency of the proposed RC system.

The findings of this study highlight the advantages of the UFLEX TFT‐based temporally reconfigurable RC system, which achieves competitive performance while substantially reducing computational complexity and processing demands. This capability suggests that the system holds strong potential as a valuable tool for supporting radiologists and clinicians in conducting faster and more accurate diagnostic assessments. Furthermore, the observed performance variations with respect to relaxation time in both the CIFAR and NIH datasets demonstrate the system's ability to adapt temporal dynamics in accordance with data‐specific characteristics. Such adaptability enables the system to maintain high classification accuracy across various relaxation times, depending on image pattern resolution.

In conclusion, the proposed temporally reconfigurable RC system provides a versatile framework for recognizing and classifying spatiotemporal data across a wide range of time scales and complexity levels.

## 3 Conclusion

In this study, we design and implement a temporally reconfigurable RC hardware based on UFLEX TFTs to overcome the performance limitations imposed by the fixed temporal dynamics of conventional hardware RC systems. The UFLEX TFTs incorporate an organic–inorganic hybrid AlO_x_ electrolyte layer, deposited via iCVD, which simultaneously functions as the gate dielectric. This design offers excellent scalability, gate controllability, and mechanical flexibility. The fabricated devices exhibit remarkable uniformity across cycles and between devices, as well as high endurance. Furthermore, leveraging an ultrathin MoS_2_ channel on a PI substrate allows the devices to maintain stable electrical performance and clear state separability even under a minimal bending radius of 3.5 mm and after 1000 repeated bending cycles. These results highlight the potential of UFLEX TFTs for applications in flexible edge computing devices. To investigate the effect of tunable temporal dynamics on classification performance, we conduct experiments on two datasets with distinct spatial and temporal characteristics. The CIFAR‐10 color object image dataset, with its low resolution, benefits from longer relaxation times, whereas the NIH chest X‐ray dataset, which features high resolution, requires shorter relaxation times. By modulating the background voltage, we achieve classification accuracies of 90.3 and 81.8%, respectively. These results demonstrate that our hardware‐based approach can achieve competitive performance using significantly fewer network parameters compared to software‐based neural networks. Overall, our findings establish that advanced RC systems can effectively process diverse spatiotemporal signals, paving the way for the development of neuromorphic computing hardware capable of handling complex temporal dynamics. While the current system demonstrates promising performance across distinct datasets, extending its evaluation to broader and more diverse spatiotemporal data remains a valuable direction for future work. Future studies may focus on integrating UFLEX RC systems into flexible system‐on‐chip platforms and optimizing device architectures for task‐specific temporal dynamics.

## 4 Experimental Section

### MoS_2_ Thin Film Synthesis

The synthesis of the MoS_2_ thin films were performed through an atmospheric pressure chemical vapor deposition (APCVD) process inside a quartz tube with a 2 inch diameter. During the process, high‐purity Ar gas was supplied as the carrier gas at a flow rate of 120 sccm under atmospheric pressure conditions of 750 Torr. Throughout the growth process, 160 mg of sulfur powder was placed in the upstream section of the furnace, whereas 2.6–2.8 mg of molybdenum trioxide (MoO_3_) powder, along with the SiO_2_/Si substrate, was positioned in the downstream section. Perylene‐3,4,9,10‐tetracarboxylic acid tetrapotassium salt (PTAS) was applied to the substrate as a seeding promoter to support the growth of MoS_2_. During the reaction, the temperatures of the upstream and downstream sections of the furnace were maintained at 320 and 830 °C, respectively.

### Transfer

In a typical transfer procedure, a polystyrene (PS) solution was prepared by dissolving PS pellets (Mw = 290 000 g m ^−1^) in toluene at a concentration of 9 g per 100 mL. The prepared PS solution was then spin‐coated onto a substrate containing the grown MoS_2_ thin film. The spin‐coating process was carried out in two steps: 500 rpm for 5 s, followed by 4500 rpm for 60 s. Subsequently, the coated sample was subjected to low‐temperature baking at 85 °C in a convection oven to facilitate solvent evaporation. For transfer, the prepared sample was placed on a flat surface, and deionized (DI) water droplets were applied to the edge of the coated PS layer or the substrate, allowing the MoS_2_ thin film to be delaminated along with the PS support layer. The detached MoS_2_ thin film (with the PS support layer) was then transferred onto the target substrate. Following the wet transfer process, the substrate underwent a two‐step baking procedure at 85 and 150 °C in a convection oven to remove residual DI water and enhance adhesion between the MoS_2_ film and the target substrate. Finally, the PS support layer was removed using toluene, followed by rinsing with DI water.

### Deposition of Hybrid AlO_x_ via iCVD

The hybrid AlO_x_ dielectric film was synthesized using an iCVD reactor. HEMA (99%, Aldrich, USA), TMA (99.99%, UP Chemical, Korea), and TBPO (99%, Aldrich, USA) were introduced into the reactor in vapor form without additional purification. To maintain stable pressure in the source injection lines, the temperatures of HEMA, TBPO, and TMA were set to 60, 30, and 30 °C, respectively. The source injection line pressures for HEMA, TBPO, and TMA were controlled at 300, 100, and 150 mTorr, respectively, to facilitate the synthesis of the organic‐inorganic hybrid film. The reaction chamber pressure and substrate temperature were maintained at 300 mTorr and 40 °C, respectively. The polymerization reaction was initiated by setting the filament temperature to 180 °C.

### Device Fabrication

A PI film was prepared for the fabrication of UFLEX TFTs. The substrate was sequentially cleaned using acetone and isopropyl alcohol in an ultrasonic bath. After cleaning, an epoxy‐based photoresist (SU‐8 2, Microchem Corporation) was spin‐coated onto the substrate and cross‐linked under UV exposure to planarize the rough PI surface, forming a 2 µm thick planarization layer. The MoS_2_ thin film was then transferred onto the prepared substrate using a previously described transfer process. To define the source and drain electrodes, Cr (5 nm)/Au (35 nm) thin films were deposited via thermal evaporation, followed by a lift‐off process for patterning. Subsequently, a 10 nm thick hybrid AlO_x_ dielectric layer, serving as an electrolyte reservoir, was deposited via iCVD. A 12 nm thick Al_2_O_3_ barrier layer was then deposited using ALD. Finally, the top‐gate electrode was formed using the same method as the source and drain electrodes.

### Electrical Characterization

The electrical characteristics of the fabricated devices were evaluated using a Keithley 4200 semiconductor parameter analyzer. All electrical measurements were conducted in a vacuum environment. DC sweep and pulse signal measurements were conducted with the Keithley 4200, integrated with the Keithley 4225‐PMU (pulse measurement unit) and 4225‐RPM (remote amplifier/switch).

## Conflict of Interest

The authors declare no conflict of interest.

## Supporting information



Supporting Information

Data File

## Data Availability

The data that support the findings of this study are available from the corresponding author upon reasonable request.
