# Supplementary material for: Temporally Reconfigurable Reservoir Computing with Flexible Electrolyte‐Gated TFTs for High‐Performance Neuromorphic Processing
Source: Adv Mater. 2025 Sep 17;38(1):e07979. doi: 10.1002/adma.202507979 (PMC12759255; doi:10.1002/adma.202507979)
Supplement: Supplementary file 1 — Supporting Information [file ADMA-38-e07979-s002.docx]

Supporting Information

Temporally Reconfigurable Reservoir Computing with Flexible Electrolyte-Gated TFTs for High-Performance Neuromorphic Processing

Kang Hyun Lee, Seohak Park, Mingu Kang, Jungyeop Oh, Wonbae Ahn, Hyeonji Lee, Seungsun Yoo, Hyunmin Kim, Min Kyu Lee, and Sung-Yool Choi*

**
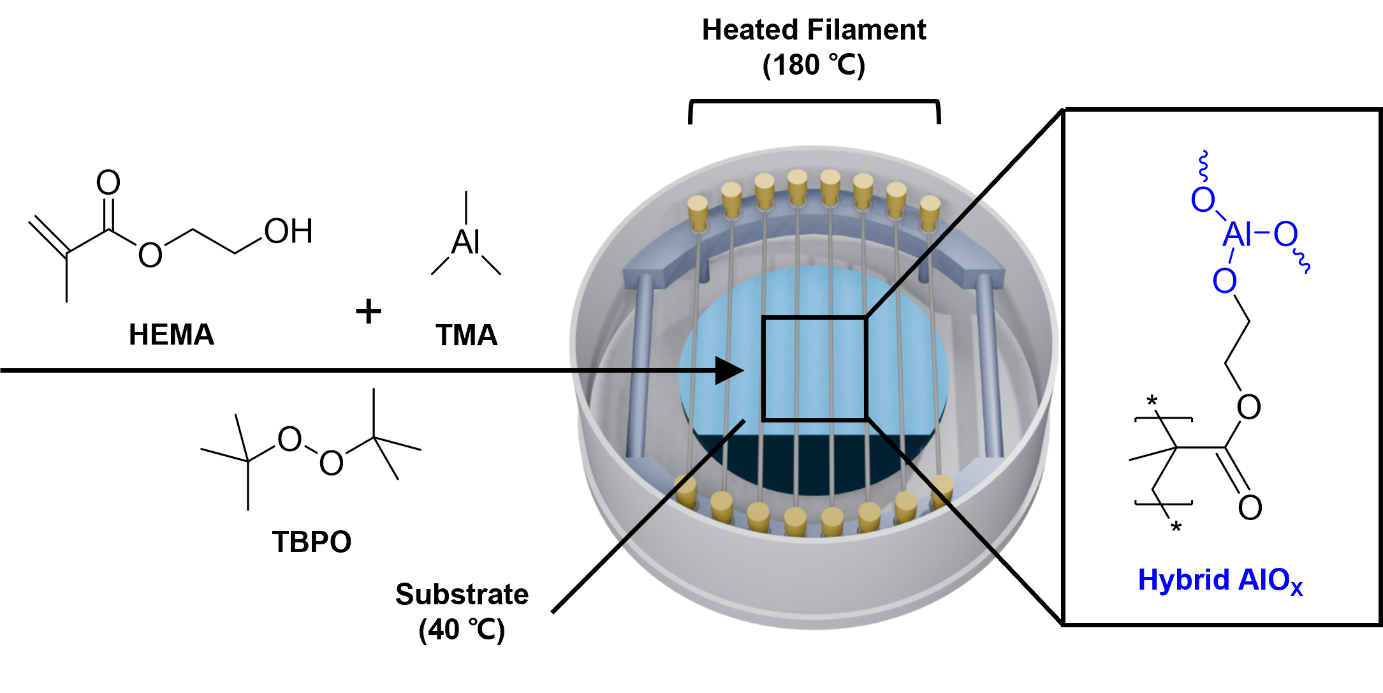
**

**Figure S1.** Schematic representation of the iCVD process, consisting of a heated filament, initiator, and monomers.


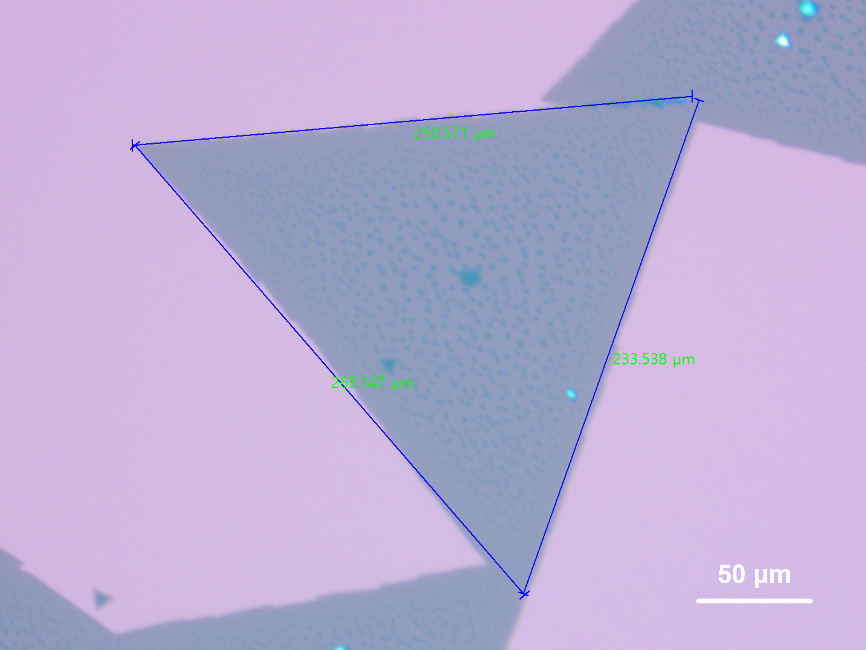


**Figure S2.** Optical image of the synthesized MoS_2_ film in a triangular form on a SiO_2_ substrate. The scale bar represents 50 μm.

**
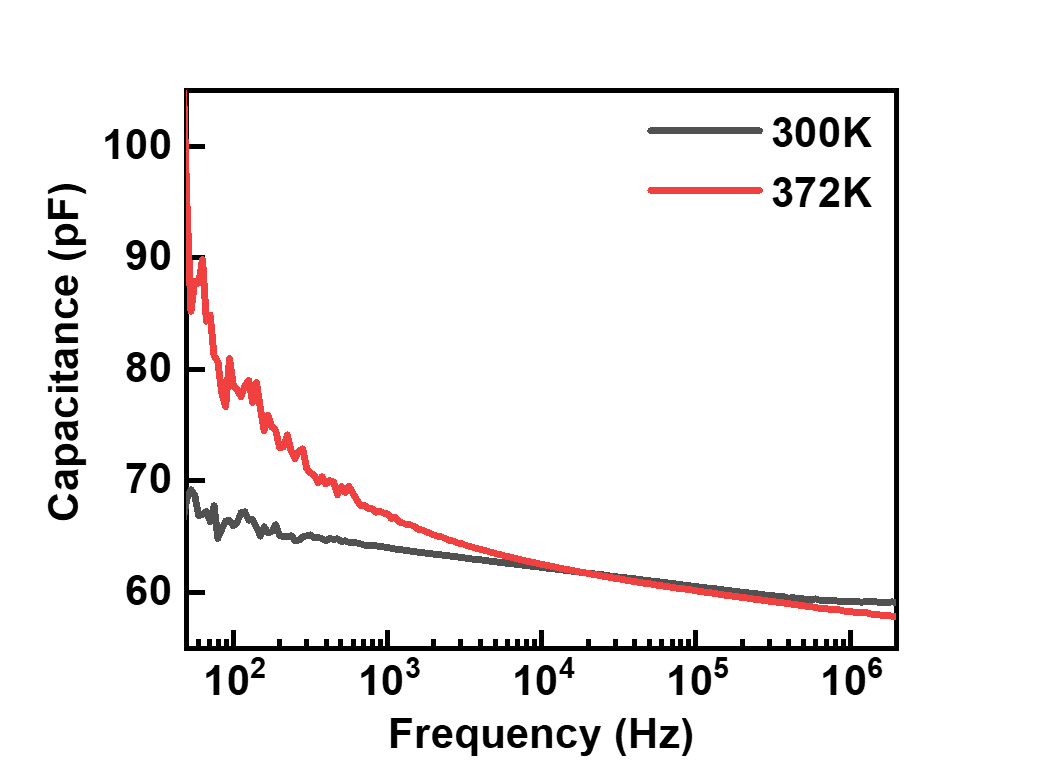
**

**Figure S3.** The frequency-capacitance characteristics of the hybrid AlO_x_ MIM capacitor investigated at temperatures of 300 K and 372 K.


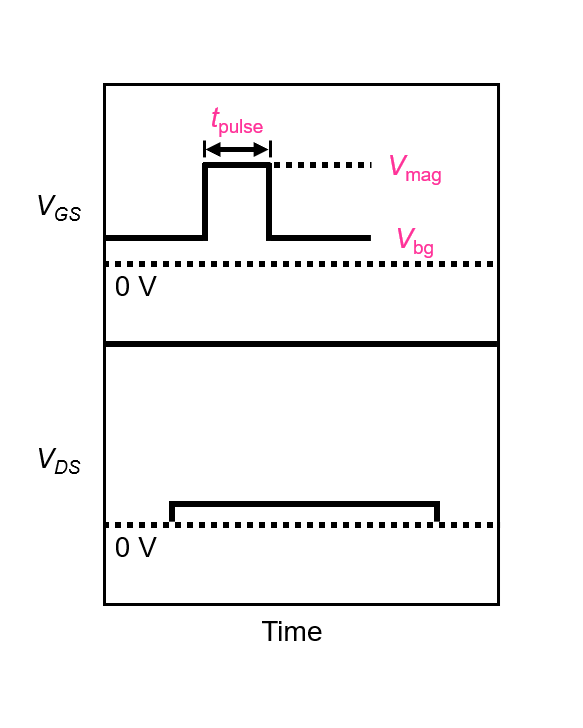


**Figure S4.** Schematic representation of the measurement pulse waveform. Square-wave voltage signal is applied to the gate electrode, while the drain electrode is biased at a constant voltage. Pulse duration (*t*_pulse_), pulse magnitude (*V*_mag_), and background voltage (*V*_bg_​) are indicated in the figure.


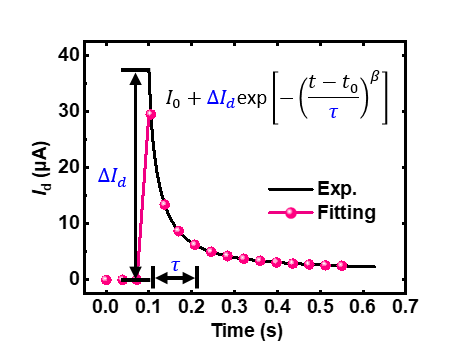


**Figure S5.** Model and the experimental result of the STM behavior. The relaxation of the drain current relaxation following a single pulse is well described by the stretched-exponential relaxation model.


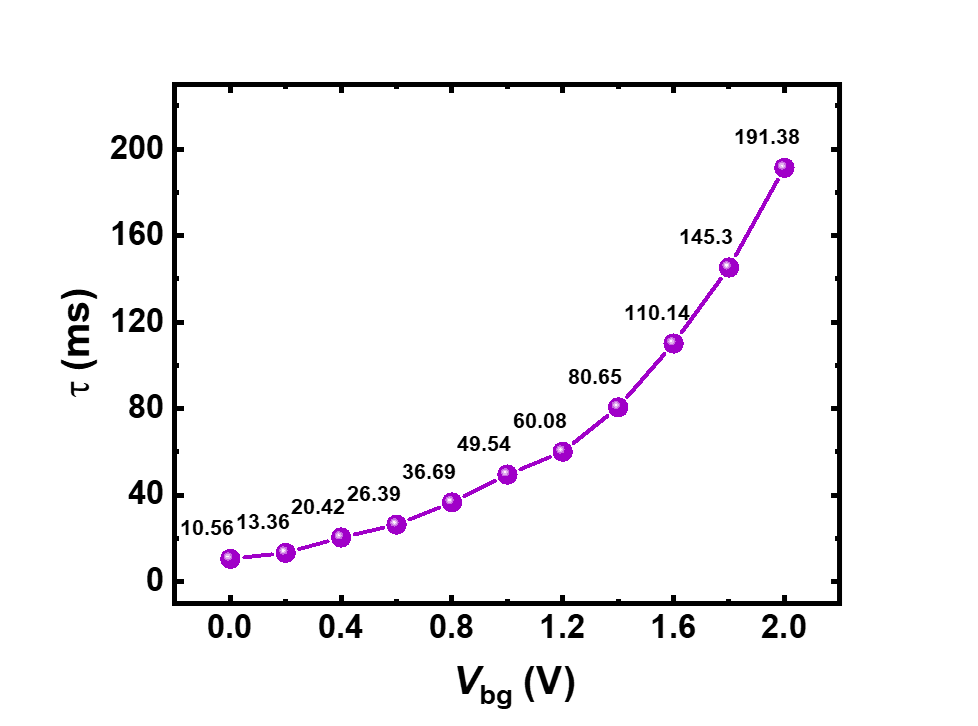


**Figure S6.** Dependence of τ on *V*_bg_. 11 repeated measurements are conducted for each *V*_bg_ under conditions of *t*_pulse_ = 30 ms, *V*_mag_ = 5 V, *V*_DS_ = 1 V. As *V*_bg_ increases from 0 to 2 V, τ increases from 10.6 ms to 191.4 ms.

**
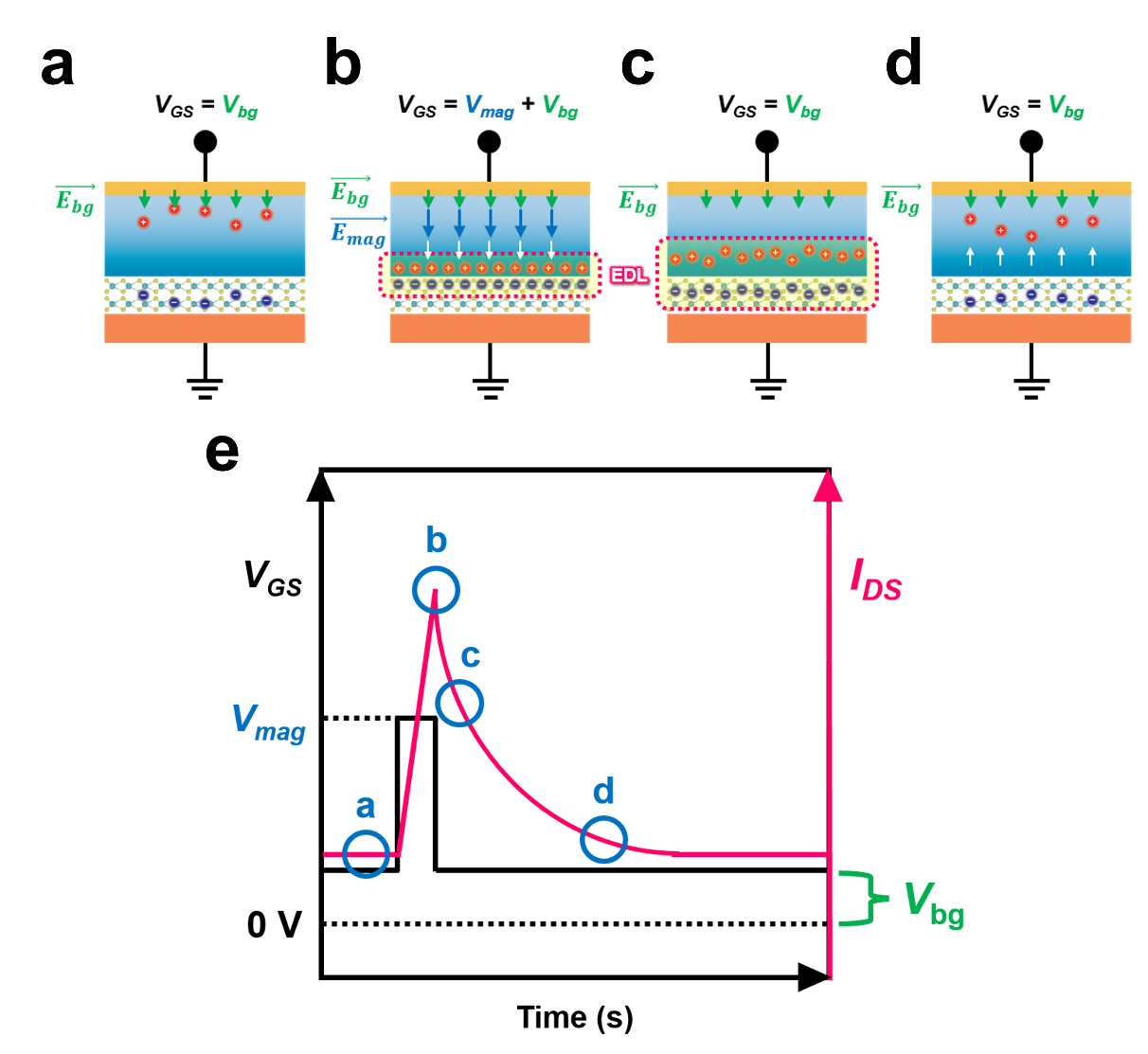
**

**Figure S7.** Drain current response of the UFLEX TFT to a gate voltage pulse superimposed on a continuously applied positive *V*_bg_. The continuous application of positive *V*_bg_ after the pulse delays proton back-diffusion, thereby sustaining the EDL and increasing the relaxation time. (a) Baseline state before the pulse is applied, where the *I*_DS_ is in equilibrium under *V*_bg_ only. (b) The moment the pulse ends, showing the peak *I*_DS_ due to EDL formation from proton accumulation near the channel. (c) Intermediate relaxation state where the pulse has ended and *I*_DS_ begins to decrease as protons gradually diffuse back into the electrolyte. (d) Near-complete relaxation state where *I*_DS_ has almost returned to the baseline. (e) Overlaid plot of the *V*_GS_ and corresponding *I*_DS_, illustrating the temporal relationship between the applied gate pulse and the current response.

**
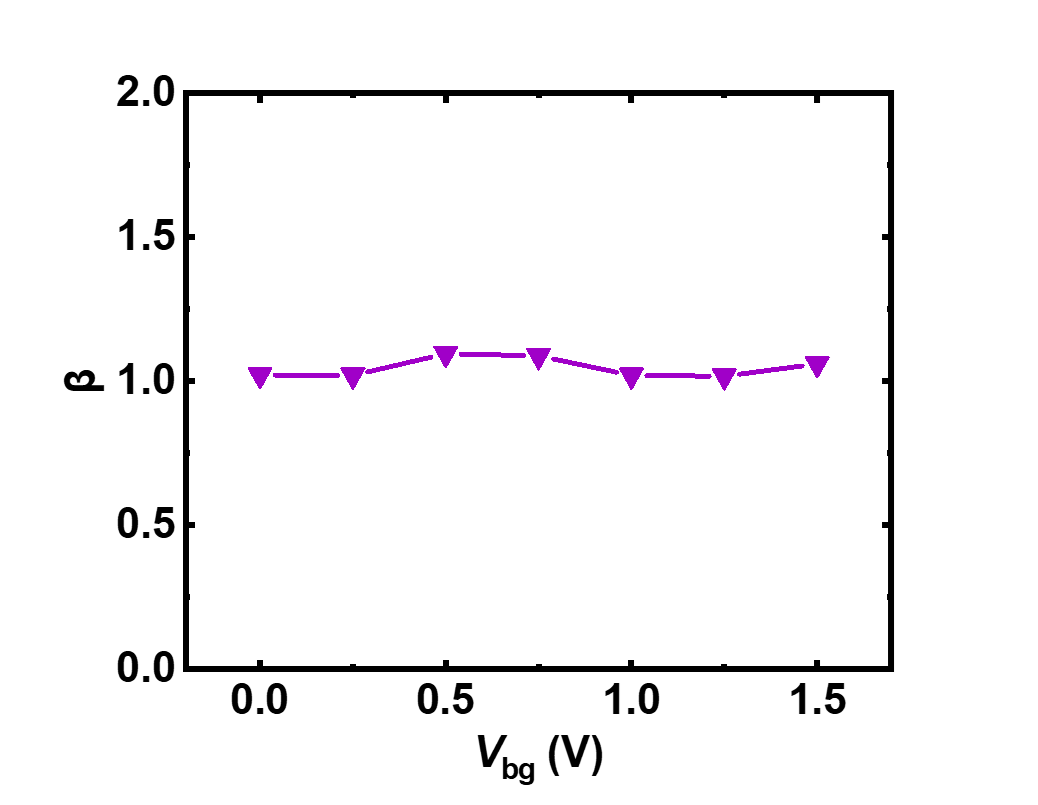
**

**Figure S8.** Dependence of β on *V*_bg_. The relationship between β and *V*_bg_ is presented, referencing the experimental data in Figure 3f. As shown, β remains nearly constant across different *V*_bg_ levels, indicating its independence from *V*_bg_ modulation. Consequently, the analysis of STM characteristics can primarily focus on τ. The measurements are conducted at *t*_pulse_ = 30 ms, *V*_mag_ = 5 V, *V*_DS_ = 1 V.

**
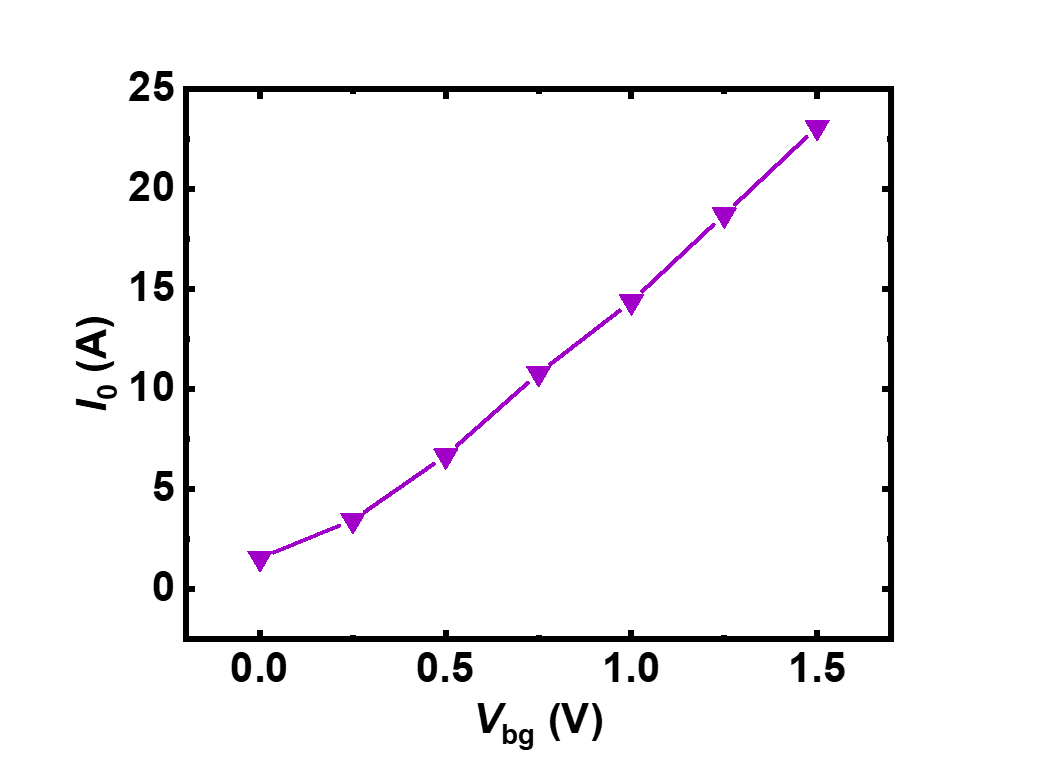
**

**Figure S9.** Dependence of the initial current state (*I*_0_) on *V*_bg_. *I*_0_–*V*_bg_ relationship is investigated over the range of *V*_bg_ = 0–1.5 V. Experimental results indicate that the initial drain current *I*_0_ increases with increasing *V*_bg_.


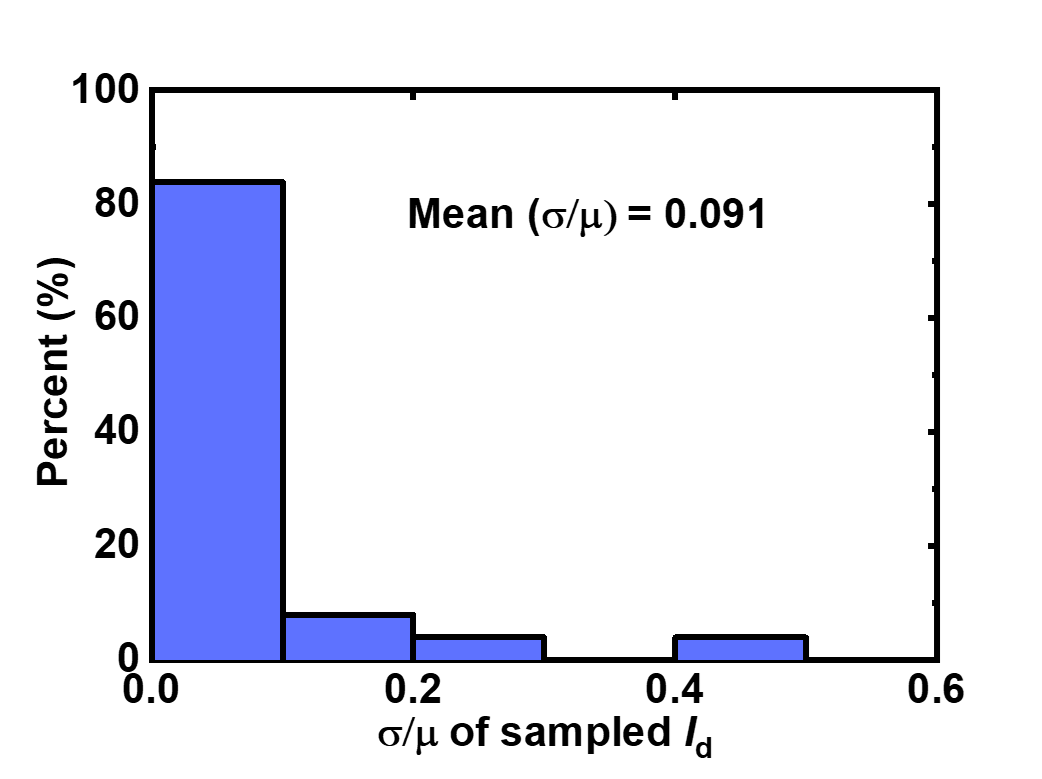


**Figure S10.** Cycle-to-cycle variation. The readout current is sampled from 25 uniformly distributed time points in figure 3g, and the variation level is quantified as σ/μ for each time point. The histogram shows that σ/μ remains within 0.1 for most time points, indicating high uniformity.


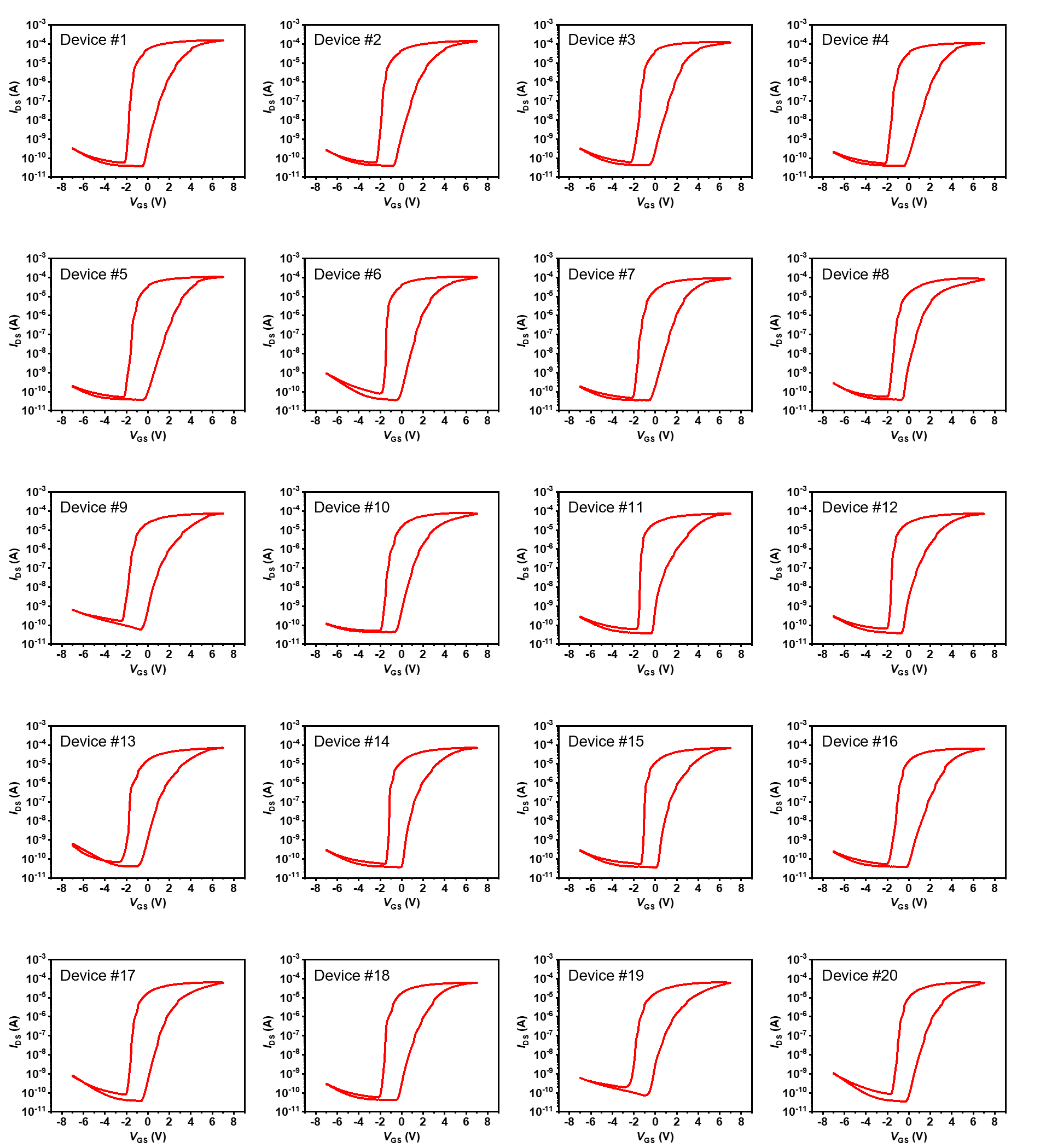


**Figure S11.** Device-to-device variation in hysteresis behavior. 20 randomly selected UFLEX TFTs are evaluated using *V*_GS_ dual sweep measurements. The results confirm that all selected devices function properly and exhibit qualitatively consistent characteristics.


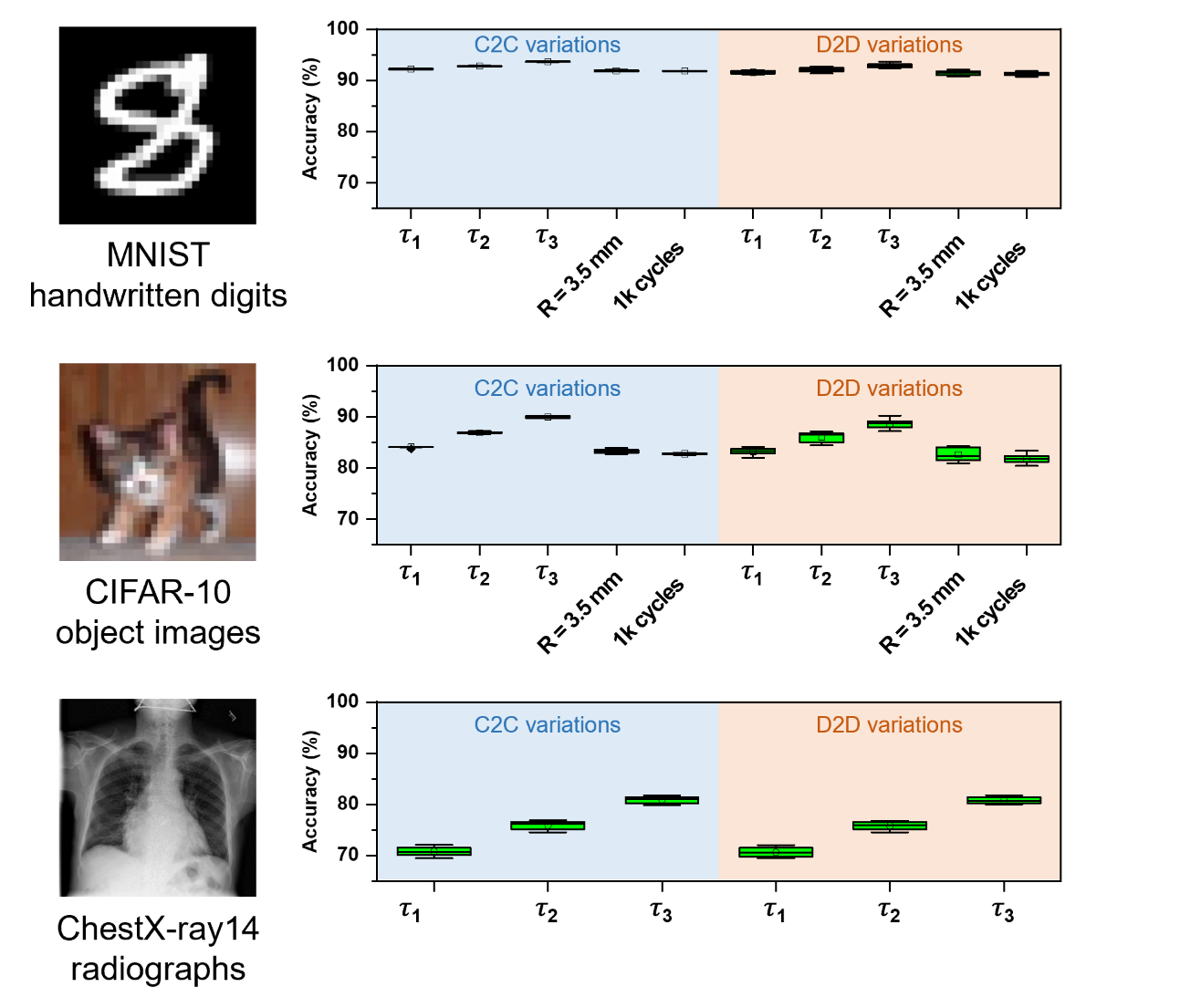


**Figure S12.** Boxplot comparison of classification accuracies under five different operating conditions: *V*_bg_ = 0, 200, and 400 mV, bending strain (R = 3.5 mm), and cyclic bending (1,000 cycles). Each boxplot summarizes the results from both cycle-to-cycle (20 repeated stimulations on a single device) and device-to-device (20 different devices) variation tests. Simulations were performed on MNIST handwritten digits, CIFAR-10 color object images, and NIH chest X-ray images. The consistently narrow accuracy distributions across all datasets and conditions confirm the system-level robustness of the UFLEX TFT-based RC platform against both C2C and D2D variation.

**
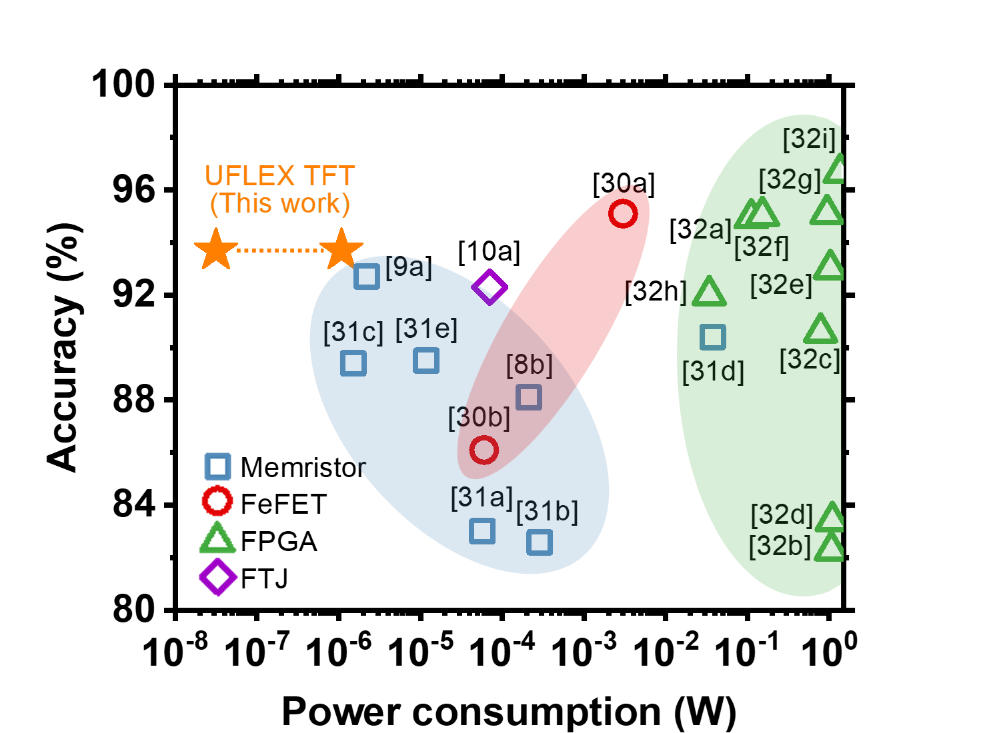
**

**Figure S13.** Power consumption versus classification accuracy (on the MNIST dataset) for various neuromorphic device platforms. Data points are obtained from previously reported studies. The UFLEX TFT achieves high accuracy (93.7%) with exceptionally low instantaneous power consumption ranging from a minimum of 31.5 nW to a maximum of 1.08 μW during actual reservoir computing operation. This unique combination of high performance and ultralow power highlights the UFLEX TFT as a highly promising platform for energy-efficient neuromorphic computing applications.

**
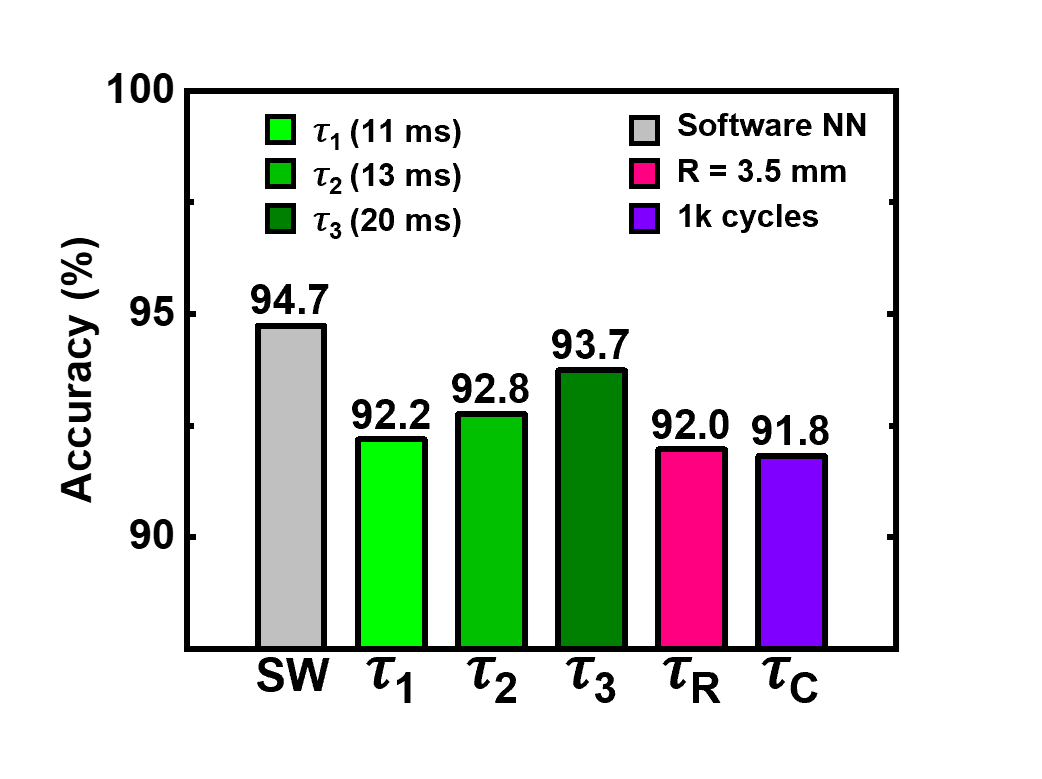
**

**Figure S14.** Comparison of experimental accuracies on the MNIST dataset across different temporal characteristics. As τ increases (τ_1_ = 11 ms, τ_2_ = 13 ms, τ_3_ = 20 ms), accuracy improves. The performance degradation of UFLEX TFTs after bending tests is negligible.


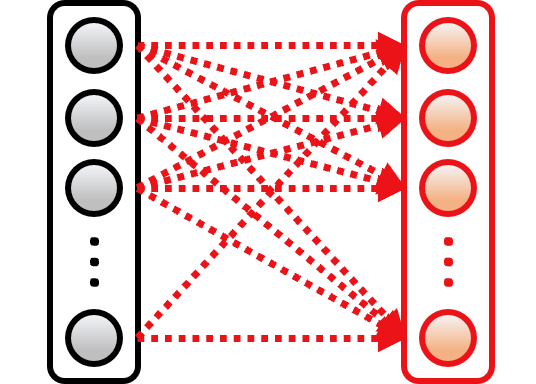


**Figure S15.** Schematic of the concept of typical neural networks without reservoir layer. Each neural layer is interconnected through a network of synaptic connections.

**
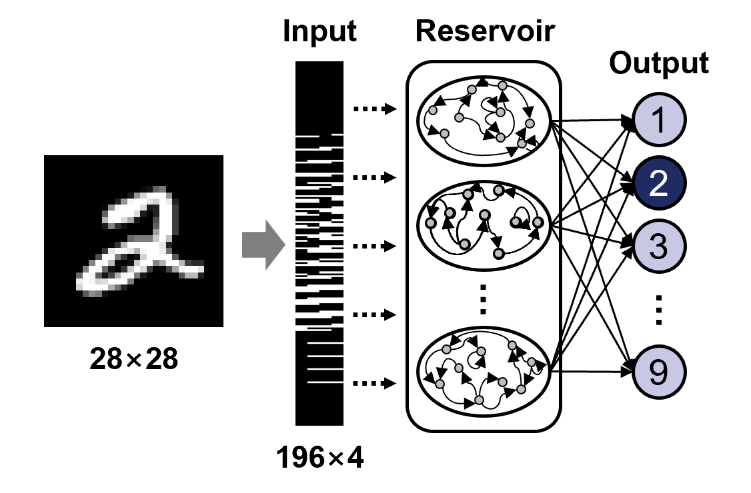
**

**Figure S16.** Process flow of 28 × 28-pixel MNIST dataset classification.

**
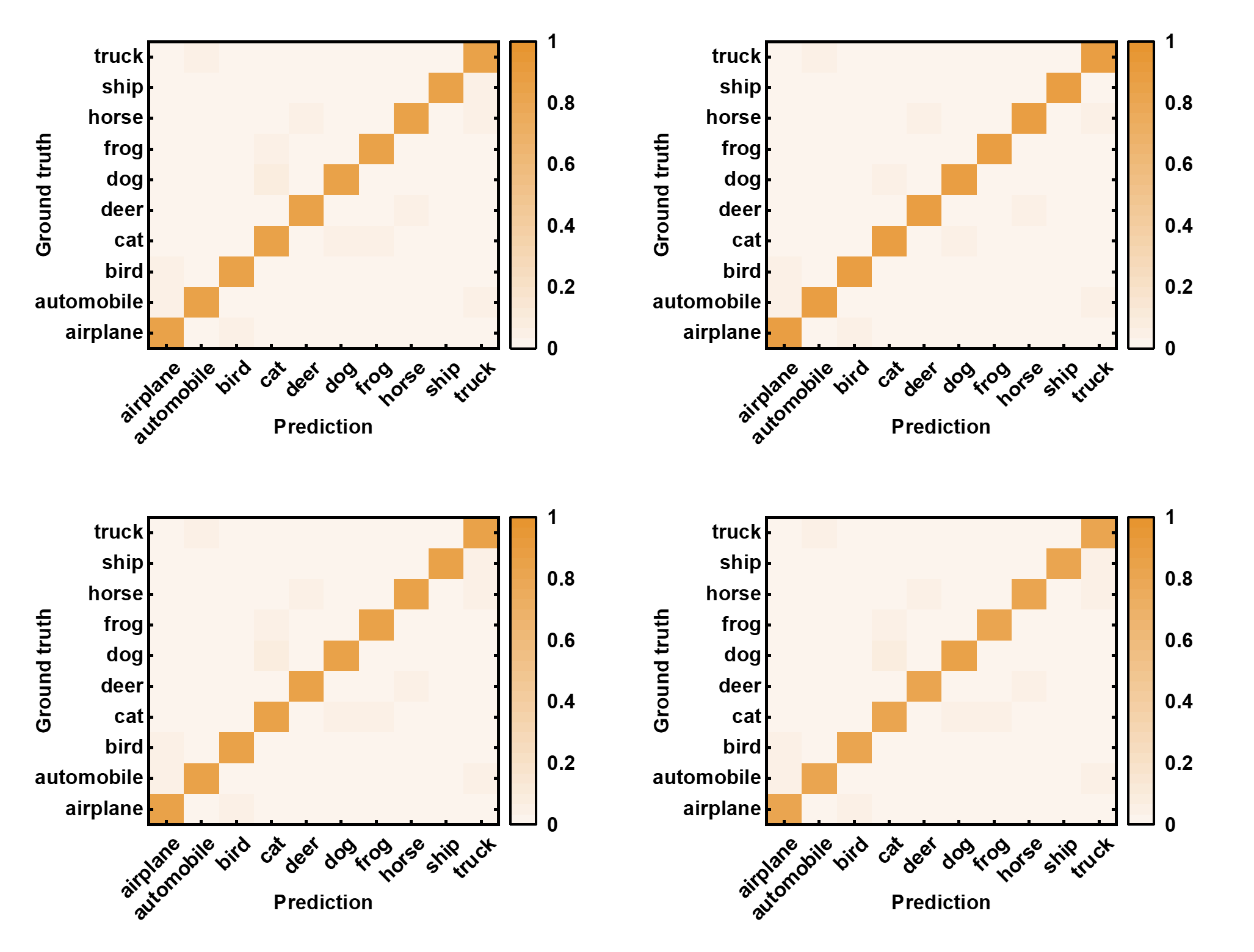
**

**Figure S17.** Confusion matrices of recognition performance on the CIFAR-10 color object image dataset. The first matrix corresponds to τ_1_, the second to τ_2_, the third to the bending strain condition with a bending radius of 3.5 mm (ε = 1.79%), and the fourth to the result after 1,000 bending cycles under 1.25% strain.

**
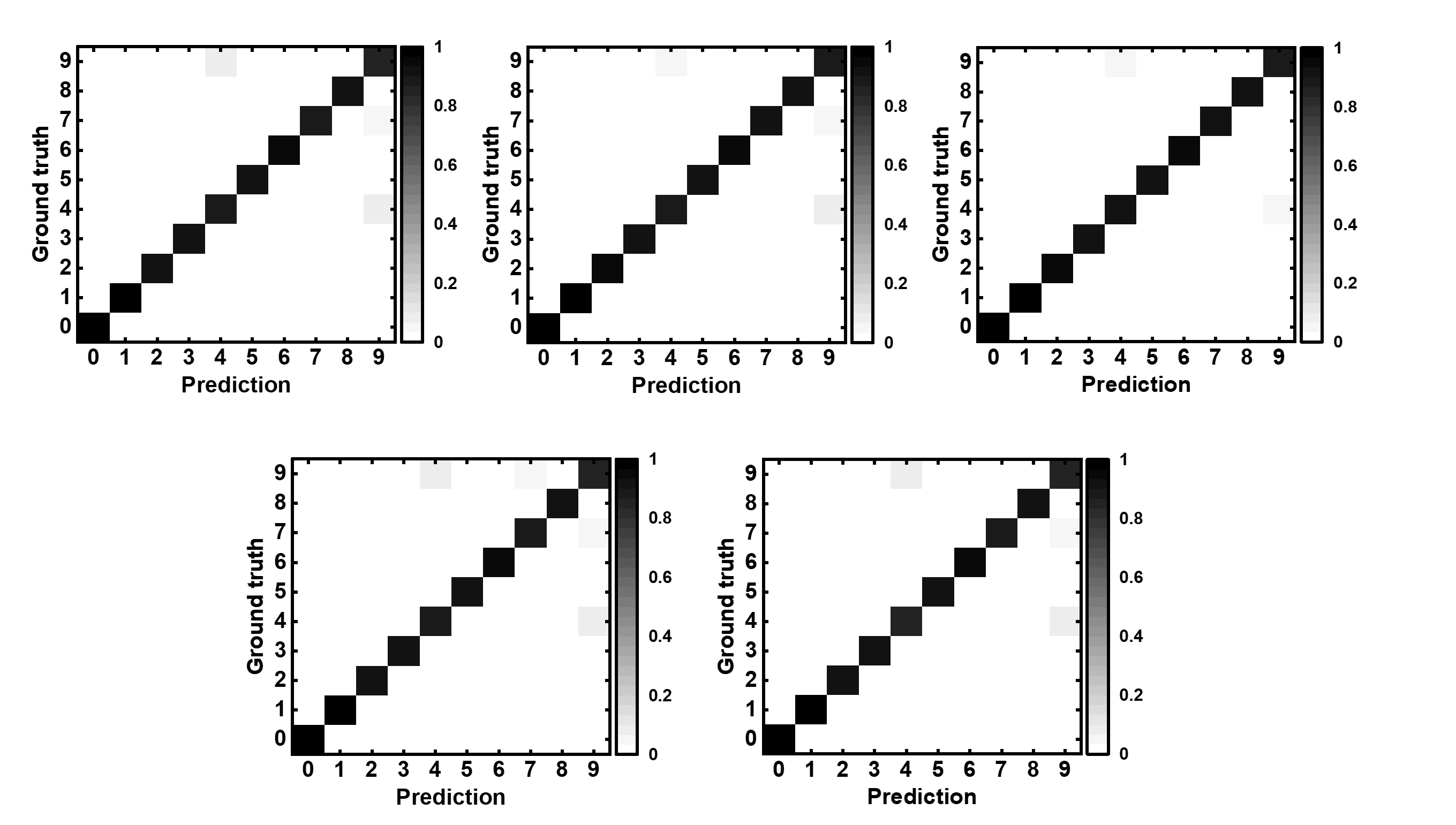
**

**Figure S18.** Confusion matrices of recognition performance on the MNIST handwritten digit dataset. The first matrix corresponds to τ_1_, the second to τ_2_, the third to τ_3_, the fourth to the bending strain condition with a bending radius of 3.5 mm (ε = 1.79%), and the fifth to the result after 1,000 bending cycles under 1.25% strain.


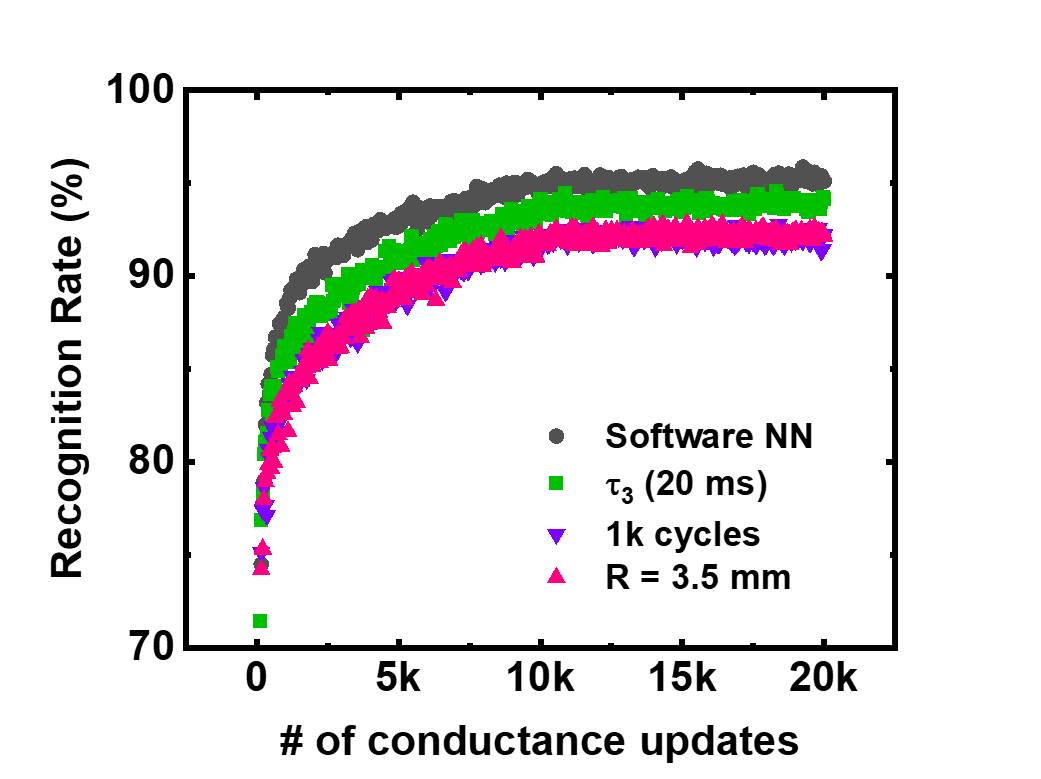


**Figure S19.** The training performance of hardware implemented temporally reconfigurable RC system with UFLEX TFT using the MNIST dataset.

**
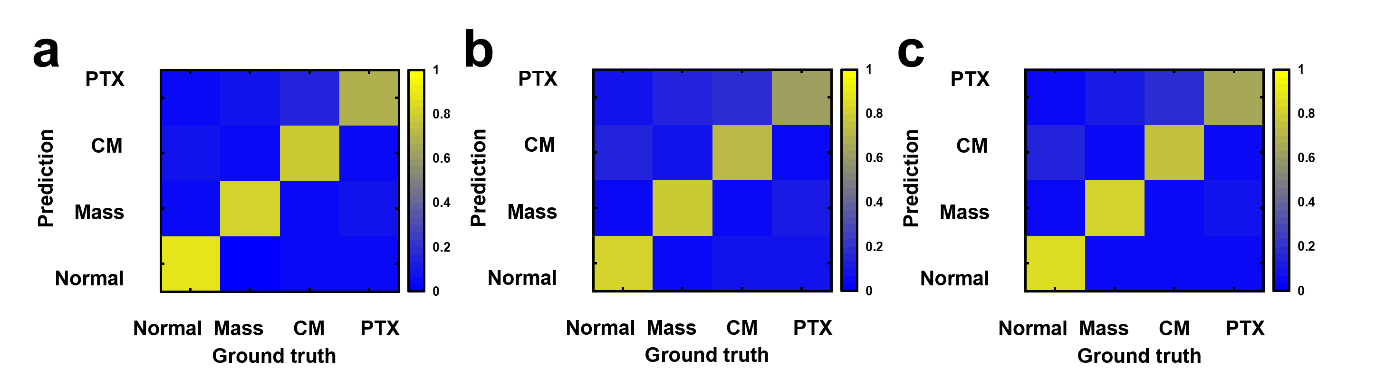
**

**Figure S20.** Confusion matrices of recognition performance on the NIH chest X-ray image dataset. The first matrix corresponds to the single-layer perceptron (SLP), while the second and third illustrate the results for τ_2_ and τ_3_, respectively.

**
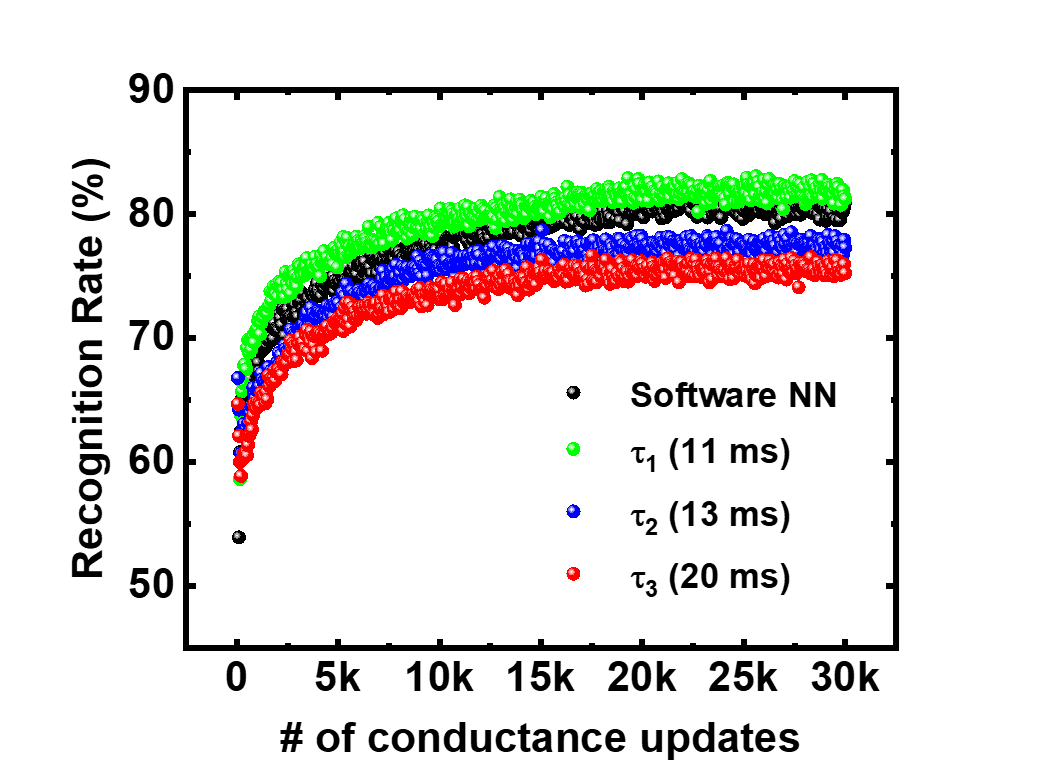
**

**Figure S21.** Training performance of the hardware-implemented RC system utilizing UFLEX TFTs. As the relaxation time of UFLEX TFTs increases, the system demonstrates enhanced performance in classifying medical images from the NIH chest X-ray dataset, indicating improved temporal information processing capability.

**Table S1.** Comparison of UFLEX TFTs with other reported electrolyte-gated TFTs.

| **TFT channel** | **Flexible substrate** | **Electrolyte** | **Strain**  **(bending radius)** | **Bending cycle** | **On/off current raio** | **Hysteresis window** | | **Accuracy** | |  |
| --- | --- | --- | --- | --- | --- | --- | --- | --- | --- | --- |
| MoS_2_  [This work] | PI  (125 μm) | Hybrid AlO_X_  (iCVD deposition) | 1.25%  (5.0 mm) | 1,000 | > 5 × 10^5^ | 2.0 V  (-7 to 7 V) | | 92.0 %  (MNIST) | |  |
|  |  |  |  |  |  |  |  | 83.9 %  (CIFAR-10) | |  |
|  |  |  | 1.79%  (3.5 mm) | - | ~ 10^6^ | 2.4 V  (-7 to 7 V) | | 83.0 %  (CIFAR-10) | |  |
|  |  |  |  |  |  |  |  | 92.0 %  (MNIST) | |  |
| MoS_2_^[15]^ | PMMA  (10 μm) | LiClO_4_  (ionic-gel) | 0.1%  (5 mm) | - | ~ 3.5 × 10^3^ | 0.8 V  (-2 to 2 V) | | - | |  |
| MoS_2_^[16]^ | PET | Al_2_O_3_/ZrO_2_/Al_2_O_3_  (ALD deposition) | -  (5.0 mm) | - | ~ 10^6^ | 3 V  (-5 to 5 V) | | - | |  |
| MoS_2_^[17]^ | PI  (20 μm) | LiSiO_X_  (sputter deposition) | 0.25%  (10 mm) | 700 | - | - | | 92.4%  (MNIST) | |  |
| Graphdiyne/MoS_2_^[18]^ | PET | LiClO_4_  (ionic-gel) | −2 ~ 2%  (2.5 mm) | 1,000 | 10^3^ | - | | 93%  (MNIST) | |  |
| ITO^[19]^ | PET  (500 μm) | Maltose-ascorbic acid  (ionic-gel) | -  (5.0 mm) | 1,000 | - | - | | - | |  |
| IGZO^[20]^ | PI  (10 μm) | HACC/PVA/DS | -  (6.0 mm) | 200 | ~ 2 × 10^4^ | 1 V  (-3 to 3 V) | | 80%  (MNIST) | |  |
| SIZO^[21]^ | PVP-coated PI | [EMIM]^+^/[TFSI]^-^  (ionic-gel) | 0.26%  (5.0 mm) | 1,500 | ~ 10^5^ | 20 V  (-20 to 20 V) | | > 90%  (Hand sign) | |  |
| VO_2_^[22]^ | Mica | [DEME][BF_4_]/  PVDF-HFP  (ionic-gel) | 0.8%  (6.0 mm) | 500 | 10^3^ | - | | 95%  (MNIST) | |  |
| Dif-TES-ADT^[23]^ | PEN  (175 μm) | PVP | 0.8%  (11 mm) | 1,000 | 4.6$\times$10^5^ | - | - | | - | |

* If the on/off current ratios or hysteresis windows were not reported in the reference, they were extracted from the transfer curves.

**Table S2.** Comparison of our reservoir system with other reported reservoir systems.

| **Device** | **Active layer/ Channel** | **Reservoir functional layer** | | **Mechanism** | **RC architecture** | **Application** | **Input size**  **(Resolution/time steps)** | **Accuracy** |
| --- | --- | --- | --- | --- | --- | --- | --- | --- |
| Electrolyte-gated transistor  [This work] | MoS_2_ | Hybrid AlO_x_ | | Ion-gating | Temporally reconfigurable RC | MNIST digits | 28 × 28 | 93.7% |
|  |  |  |  |  |  | CIFAR-10  object images | 32 × 32 × 3 | 90.3% |
|  |  |  |  |  |  | ChestX-ray14 radiographs | 256 × 256  (Resized) | 81.8% |
| Charge trap memory^[1]^ | MoS_2_ | Te | | Charge trap | Single | N-MNIST digits | 34 × 34 | 90.8% |
| Charge trap memory^[2]^ | MoS_2_ | PHPS-based CTL | | Charge trap | Temporally reconfigurable RC | MNIST digits | 28 × 28 | 91.0% |
| Charge trap memory^[3]^ | MoS_2_ | SiO_2_ | | Charge trap | Single | Monochrome images of digits | 5 × 4 | 95.5% |
| Ferroelectric FET^[4]^ | 𝛼-In_2_Se_3_ | 𝛼-In_2_Se_3_ | | Ferroelectric | Temporally reconfigurable RC | Modified MNIST | 20 × 20 | 86.1% |
| FET^[5]^ | p-NDI | | | Exciton dissociation | Single | MNIST digits | 28 × 28 | 88.2% |
| Memcapacitor^[6]^ | HZO | | | Polarization and charge trap | Single | MNIST digits | 28 × 28 | 86.2% |
|  |  |  |  |  |  | putEMG hand gestures | - | 94.3% |
| Photo-synapse^[7]^ | a-GaO_x_ | | | Persistent photoconductivity | Single | FVC 2002 Fingerprint | 20 × 4 | > 90% |
| Memristor^[8]^ | MoS_2_ | | | Metal-ion transition | Single | MNIST digits | 28 × 28 | 86.7% |
| Memristor^[9]^ | BFO/SRO | | | Polarization | Single | Monochrome images of digits | 5 × 3 | 89.5% |
| Memristor^[10]^ | h-BN | | | Electrochemical metallization | Single | MNIST digits | 28 × 28 | 92.7% |
| Memristor^[11]^ | WO_x_ | | | Valence change mechanism | Single | MNIST digits | 28 × 28 | 88.1% |
| Memristor^[12]^ | P3HT@TDA-PW | | | Electrochemical metallization and charge trapping | Single | Emotion | 20 × 20 | 91.2% |
|  |  |  |  |  |  | Modified MNIST | 28 × 28 | 89.4% |
| Memristor^[13]^ | PVP@Ag NW | | | Electrochemical metallization | Single | MNIST digits | 28 × 28 | 90.4% |
| Moiré synaptic transistor^[14]^ | BLG | | BLG/h-BN | Electronic ratcheting | Single | MNIST digits | 28 × 28 | > 90% |
|  |  |  |  |  |  | Input-specific adaptation for associative learning | - | 100% |

**Table S3.** Comparison of our reservoir system with previously reported hardware-based neuromorphic systems for CIFAR-10 classification.

| **Device** | **Active layer** | **Mechanism** | **Network architecture** | **Application** | **Accuracy** |
| --- | --- | --- | --- | --- | --- |
| Electrolyte-gated transistor  [This work] | MoS_2_ | Ion-gating | Temporally reconfigurable RC | CIFAR-10 images | 90.3% |
| Memristor^[24]^ | Ta_2_O_3_ | Valence change mechanism | Temporally reconfigurable RC | CIFAR-10 images | 88% |
| Memristor^[25]^ | - | Valence Change Mechanism | CNN | CIFAR-10 images | 84.38% |
| Memristor^[26]^ | LaAlO_3_ | Valence Change Mechanism | DNN | CIFAR-10 images | 88.1% |
| Memristor^[27]^ | TiO_2_ | VTEAM model | CNN | CIFAR-10 images | 85.42% |
| Memristor^[28]^ | SnS | Electrochemical metallization and filamentary switching | CNN | CIFAR-10 images | 89.3% |

**Supplementary References**

[1] J. Zha, Y. Xia, S. Shi, H. Huang, S. Li, C. Qian, H. Wang, P. Yang, Z. Zhang, Y. Meng, W. Wang, Z. Yang, H. Yu, J. C. Ho, Z. Wang, C. Tan, *Adv. Mater.* **2024**, 36, 2308502.

[2] H. Lee, J. Oh, W. Ahn, M. Kang, S. Park, H. Kim, S. Yoo, B. C. Jang, S.-Y. Choi, *Adv. Funct. Mater.* **2025**, 35, 2416811.

[3] M. Farronato, P. Mannocci, M. Melegari, S. Ricci, C. M. Compagnoni, D. Ielmini, *Adv. Mater.* **2023**, 35, 2205381.

[4] K. Liu, T. Zhang, B. Dang, L. Bao, L. Xu, C. Cheng, Z. Yang, R. Huang, Y. Yang, *Nat. Electron.* **2022**, 5, 761.

[5] X. Wu, S. Wang, W. Huang, Y. Dong, Z. Wang, W. Huang, *Nat. Commun.* **2023**, 14, 468.

[6] M. Pei, Y. Zhu, S. Liu, H. Cui, Y. Li, Y. Yan, Y. Li, C. Wan, Q. Wan, *Adv. Mater.* **2023**, 35, 2305609.

[7] Z. Zhang, X. Zhao, X. Zhang, X. Hou, X. Ma, S. Tang, Y. Zhang, G. Xu, Q. Liu, S. Long, *Nat. Commun.* **2022**, 13, 6590.

[8] Y. Lee, Y. Huang, Y.-F. Chang, S. J. Yang, N. D. Ignacio, S. Kutagulla, S. Mohan, S. Kim, J. Lee, D. Akinwande, S. Kim, *ACS Nano* **2024**, 18, 14327.

[9] Z. Chen, W. Li, Z. Fan, S. Dong, Y. Chen, M. Qin, M. Zeng, X. Lu, G. Zhou, X. Gao, J.-M. Liu, *Nat. Commun.* **2023**, 14, 3585.

[10] W. Ahn, S. Lee, J. Oh, H. Lee, S.-Y. Choi, *Adv. Mater.* **2025**, 37, 2413640.

[11] C. Du, F. Cai, M. A. Zidan, W. Ma, S. H. Lee, W. D. Lu, *Nat. Commun.* **2017**, 8, 2204.

[12] G. Zhang, Z. Y. Xiong, Y. Gong, Z. Zhu, Z. Lv, Y. Wang, J. Q. Yang, X. Xing, Z. P. Wang, J. Qin, *Adv. Funct. Mater.* **2022**, 32, 2204721.

[13] G. Milano, G. Pedretti, K. Montano, S. Ricci, S. Hashemkhani, L. Boarino, D. Ielmini, C. Ricciardi, Nat. Mat. 2022, 21, 195.

[14] X. Yan, Z. Zheng, V. K. Sangwan, J. H. Qian, X. Wang, S. E. Liu, K. Watanabe, T. Taniguchi, S.-Y. Xu, P. Jarillo-Herrero, Nature 2023, 624, 551.

[15] X. Gao, J. Yin, J. Zhu, J. Chang, J. Zhang, Y. Hao, *IEEE Electron Device Lett.* **2024**, 45, 605.

[16] T. Y. Wang, J. L. Meng, Z. Y. He, L. Chen, H. Zhu, Q. Q. Sun, S. J. Ding, P. Zhou, D. W. Zhang, *Adv. Sci.* **2020**, 7, 1903480.

[17] Y. Hwang, B. Park, S. Hwang, S. W. Choi, H. S. Kim, A. R. Kim, J. W. Choi, J. Yoon, J. D. Kwon, Y. Kim, *Small Methods* **2023**, 7, 2201719.

[18] B. W. Yao, J. Li, X. D. Chen, M. X. Yu, Z. C. Zhang, Y. Li, T. B. Lu, J. Zhang, *Adv. Funct. Mater.* **2021**, 31, 2100069.

[19] W. Qin, B. H. Kang, H. J. Kim, *ACS Appl. Mater. Interfaces* **2021**, 13, 34597.

[20] L. Yuan, T. Zhao, J. Dai, L. Xue, X. Zhang, C. Peng, P. Wen, H. Liu, H. Hu, L. Chen, H. Xin, J. Li, X. Li, J. Zhang, *Adv. Funct. Mater.* **2025**, 2418052.

[21] S. Oh, J.-I. Cho, B. H. Lee, S. Seo, J.-H. Lee, H. Choo, K. Heo, S. Y. Lee, J.-H. Park, *Sci. Adv.* **2021**, 7, eabg9450.

[22] X. Deng, S. Q. Wang, Y. X. Liu, N. Zhong, Y. H. He, H. Peng, P. H. Xiang, C. G. Duan, *Adv. Funct. Mater.* **2021**, 31, 2101099.

[23] J. Shi, J. Jie, W. Deng, G. Luo, X. Fang, Y. Xiao, Y. Zhang, X. Zhang, X. Zhang, *Adv. Mater.* **2022**, 34, 2200380.

[24] F. Nowshin, Y. Huang, M. R. Sarkar, Q. Xia, Y. Yi, *IEEE Transactions on Circuits and Systems I: Regular Papers* **2023**, 71, 174.

[25] H. Ran, S. Wen, Q. Li, Y. Yang, K. Shi, Y. Feng, P. Zhou, T. Huang, *IEEE Transactions on Neural Networks and Learning Systems* **2020**, 33, 2121.

[26] T. V. Nguyen, J. An, K.-S. Min, *Micromachines* **2021**, 12, 791.

[27] K. Ravikumar, R. Sukumar, *High-Confidence Computing* **2022**, 2, 100085.

[28] X. F. Lu, Y. Zhang, N. Wang, S. Luo, K. Peng, L. Wang, H. Chen, W. Gao, X. H. Chen, Y. Bao, *Nano letters* **2021**, 21, 8800.
